# Identification of growth hormone receptor as a relevant target for precision medicine in low‐EGFR expressing glioblastoma

**DOI:** 10.1002/ctm2.939

**Published:** 2022-07-08

**Authors:** Maïté Verreault, Irma Segoviano Vilchis, Shai Rosenberg, Nolwenn Lemaire, Charlotte Schmitt, Jérémy Guehennec, Louis Royer‐Perron, Jean‐Léon Thomas, TuKiet T. Lam, Florent Dingli, Damarys Loew, François Ducray, Sophie Paris, Catherine Carpentier, Yannick Marie, Florence Laigle‐Donadey, Audrey Rousseau, Natascha Pigat, Florence Boutillon, Franck Bielle, Karima Mokhtari, Stuart J. Frank, Aurélien de Reyniès, Khê Hoang‐Xuan, Marc Sanson, Vincent Goffin, Ahmed Idbaih

**Affiliations:** ^1^ Sorbonne Université Institut du Cerveau – Paris Brain Institute – ICM, Inserm, CNRS, AP‐HP, Hôpital de la Pitié Salpêtrière Paris France; ^2^ Laboratory for Cancer Computational Biology & Gaffin Center for Neuro‐Oncology Hadassah – Hebrew University Medical Center Jerusalem Israel; ^3^ DMU Neurosciences Service de Neurologie 2‐Mazarin Sorbonne Université Institut du Cerveau – Paris Brain Institute – ICM, Inserm, CNRS, AP‐HP, Hôpital de la Pitié Salpêtrière Paris France; ^4^ Department of Neurology Yale University School of Medicine New Haven Connecticut USA; ^5^ Mass Spectrometry & Proteomics Resource, Keck Biotechnology Resource Laboratory New Haven Connecticut USA; ^6^ Department of Molecular Biophysics and Biochemistry Yale University New Haven Connecticut USA; ^7^ Institut Curie, Centre de Recherche, PSL Research University, Laboratoire de Spectrométrie de Masse Protéomique Paris France; ^8^ Hôpital Neurologique, Service de Neurologie B Lyon France; ^9^ Université Paris Cité INSERM UMR‐S1151, CNRS UMR‐S8253, Institut Necker Enfants Malades Paris France; ^10^ Division of Endocrinology, Diabetes, and Metabolism, Department of Medicine University of Alabama Birmingham Alabama USA; ^11^ Endocrinology Section, Medical Service Birmingham VA Medical Center Birmingham Alabama USA; ^12^ Programme Cartes d'Identité des Tumeurs (CIT) Ligue Nationale Contre le Cancer Service de Bioinformatique Paris France

**Keywords:** cell migration, comparative analysis, glioblastoma, oncogenicity, pre‐clinical models, therapeutic target, tumour invasion

## Abstract

**Objective:**

New therapeutic approaches are needed to improve the prognosis of glioblastoma (GBM) patients.

**Methods:**

With the objective of identifying alternative oncogenic mechanisms to abnormally activated epidermal growth factor receptor (EGFR) signalling, one of the most common oncogenic mechanisms in GBM, we performed a comparative analysis of gene expression profiles in a series of 54 human GBM samples. We then conducted gain of function as well as genetic and pharmocological inhibition assays in GBM patient‐derived cell lines to functionnally validate our finding.

**Results:**

We identified that growth hormone receptor (GHR) signalling defines a distinct molecular subset of GBMs devoid of *EGFR* overexpression. *GHR* overexpression was detected in one third of patients and was associated with low levels of suppressor of cytokine signalling 2 (*SOCS2*) expression due to *SOCS2* promoter hypermethylation. In GBM patient‐derived cell lines, *GHR* signalling modulates the expression of proteins involved in cellular movement, promotes cell migration, invasion and proliferation in vitro and promotes tumourigenesis, tumour growth, and tumour invasion in vivo. *GHR* genetic and pharmacological inhibition reduced cell proliferation and migration in vitro.

**Conclusion:**

This study pioneers a new field of investigation to improve the prognosis of GBM patients.

## Graphical abstract

- GHR overexpression is observed in one third of GBM patients.
- GHR overexpression is associated with SOCS2 promoter hypermethylation leading to low levels of SOCS2 expression.
- GHR signalling promotes cell migration, invasion and proliferation in vitro and promotes tumourigenesis, tumour growth and tumour invasiveness in vivo.
- Genetic and pharmacological GHR inhibition reduces cell proliferation and migration in vitro.

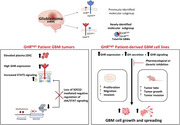

## 1 INTRODUCTION

Glioblastoma (GBM) is the most frequent primary brain cancer in adults, accounting for ∼50% of gliomas.^1^, ^2^, ^3^ In most cases, the outcome of GBM patients remains dismal with a median overall survival (OS) ranging between 12 and 24 months despite intensive treatments, including surgical resection, cytotoxic chemotherapy and radiation therapy.^4^, ^5^, ^6^ Therefore, new therapeutic approaches are needed to improve the prognosis of GBM patients.

Within the past two decades, OMICS technologies have allowed identification of recurrent molecular abnormalities and altered intracellular signalling pathways in GBM and have improved our understanding of oncogenic drivers in these tumours at the biological and clinical levels.^7^, ^8^, ^9^, ^10^ The integration of such molecular information with mechanistic and clinical data has contributed to portray a comprehensive view of GBM molecular landscape and its impact on tumour cell phenotypes and clinical behaviour.^11^, ^12^, ^13^ Importantly, these studies have led to the development of new therapeutic strategies targeting oncogenic signalling pathways for the treatment of GBM patients. Recent studies have shown the added value of patient stratification to improve targeted drug efficacy.^14^ For instance, receptor tyrosine kinase inhibitors combined with careful patient stratification has shown some efficacy for GBM treatment in clinical trials.^15^ These promising results illustrate that a better understanding of GBM oncogenic biological mechanisms is a critical step towards the development of innovative therapeutic strategies.

Epidermal growth factor receptor (*EGFR*) overexpression has been long known as the most frequent genetic alteration in GBM.^13^ It is observed in more than 60% of cases,^16^ generally as a result of *EGFR* gene amplification. However, a significant subgroup of GBMs does not present *EGFR* overexpression, yet is still linked with aggressive disease and poor prognosis. Molecular alterations present in low‐*EGFR*‐expressing GBMs have been identified (e.g. *PTEN* deletion, alterations in the *TP53* or *RB1* pathways),^13^ but none of these alterations are specific to this subgroup. Dissection of molecular alterations in these GBMs is thus important both to improve our understanding of GBM oncogenesis and to design new targeted therapies for this subgroup.

With this objective, we have conducted a molecular analysis in a panel of 54 GBM samples. This approach allowed us to identify the growth hormone receptor (GHR) pathway as an alternative oncogenic axis in low‐*EGFR*‐expressing GBMs. Indeed, functional in vitro and in vivo assays using our patient‐derived cellular models show that GHR signalling is involved in GBM cell migration and invasiveness. Enhanced migration/invasion is a major obstacle for efficient surgical resection in GBM, tumour cells dispersed in the parenchyma being a primary source of tumour relapse.^17^ We are thus establishing here GHR as an important oncogene with relevant therapeutic implications in a subgroup of GBM.

## 2 RESULTS

### 2.1 Identification of GHR signalling pathway activation in a subgroup of low‐EGFR‐expressing GBMs

**FIGURE 1:**
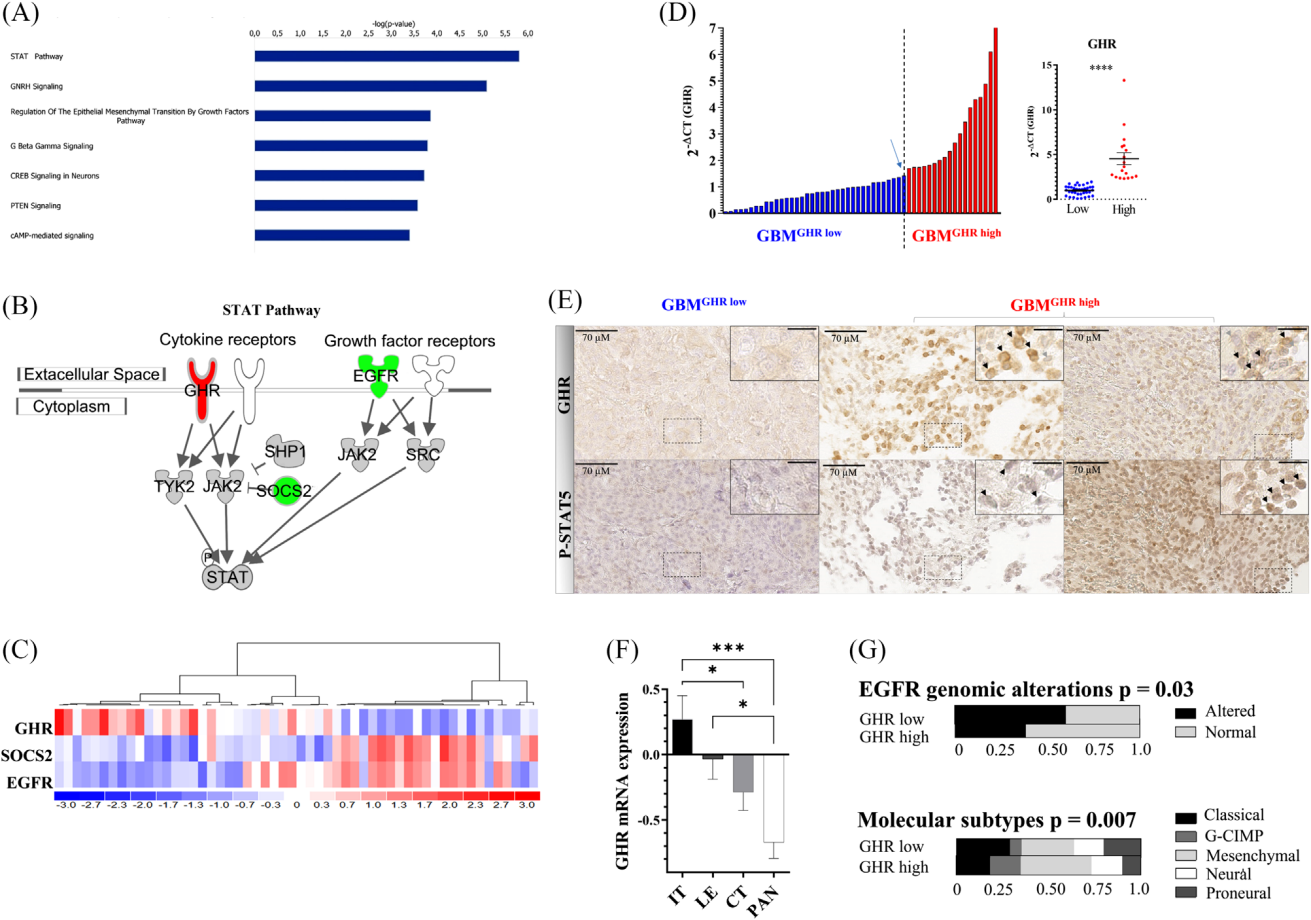
Newly diagnosed glioblastomas (GBMs) exhibit different epidermal growth factor receptor (EGFR), suppressor of cytokine signalling 2 (SOCS2) and growth hormone receptor (GHR) expression patterns. (A) Top‐most significantly modulated canonical pathways between low (*n* = 27) versus high (*n* = 27) *EGFR*‐expressing GBM. The bar size represents the –log(*p*). (B) Signal transducer and activator of transcription (STAT) signalling pathway with differential expression status (up/red, down/green) in low versus high‐*EGFR*‐expressing GBMs. (C) *EGFR*, *SOCS2* and *GHR* expression heatmap in GBM samples (*n* = 54). (D) *GHR* expression in the ONT GBM cohort measured by RT‐qPCR. A break (arrow) can be seen in the overall distribution and was used to define GBM^GHR high^ versus GBM^GHR low^ groups. Comparison of expression of *GHR* RT‐qPCR data in GBM^GHR high^ (*n* = 18) versus GBM^GHR low^ (*n* = 36). *Y*‐axis represents 2^−Δ^ *^CT^* for each gene relative to *PPIA* expression. ****p ≤ .0001. (E) Immunohistochemistry (IHC) staining of GHR (top) and p‐STAT5 (bottom) shows high‐GHR protein expression in two GBM^GHR high^ cases, whereas no protein expression is detected in a GBM^GHR low^ case. Scale bars in insets correspond to 30 μM. Black arrows show positive cells, whereas grey arrows show negative cells. Additional regions per cases and negative controls can be seen in Figure S3. GHR IHC images shown are representative of 17 GBM cases (6 GBM^GHR high^, 10 GBM^GHR low^) analysed. Positive GHR protein expression is found in ≥30% of tumour area of GBM^GHR high^ cases. (F) Distribution of GHR mRNA expression (normalized gene level RNA‐sequencing expression data in FPKM) in tumour anatomic structures defined as part of the Ivy Glioblastoma Atlas project,^73^ such as infiltrative tumour (IT), leading edge (LE), cellular tumour (CT) and pseudopalisading cells around necrosis (PAN). ***p ≤ .001; *p ≤ .05. (G) Proportional distribution of EGFR genomic alterations (mutations or amplification) and molecular subtypes in GBM^GHR high^ (*n* = 60) versus GBM^GHR low^ (*n* = 456) (TCGA dataset, *z*‐score of 1.3). Results were analysed by Pearson's chi‐square test.

Gene expression profiles of 54 supratentorial newly diagnosed de novo GBMs retrieved from the OncoNeuroTek biobank (ONT, Paris Brain Institute, Paris) were compared using data from expression microarrays. The study cohort included 37 men and 17 women (sex ratio = 2.17). The median age at diagnosis was 58.25 years (range: 26–84‐year old). *EGFR* expression distribution and *EGFR* high versus low subgroup definition can be seen in Figure S1A. Ingenuity pathway analysis (IPA) of differentially expressed genes (DEGs) between the two subgroups (Tables S1 and S2) identified signal transducer and activator of transcription (STAT) pathway as the most significantly (*p* = 1.56E − 6) modulated canonical pathway in low‐*EGFR*‐expressing GBMs (Figure 1A). As shown in Figure 1B, STAT signalling pathway per se included three DEGs: *EGFR* (down, *p* = 1.52E − 20; Tables S1 and S2) and two master regulators of GHR signalling pathway, *GHR* (upregulation *p* = 8.18E − 4) and suppressor of cytokine signalling 2 (*SOCS2*; down, *p* = 1.01E − 5). The expression of STAT genes was not different between the subgroups. Based on the expression level of these three DEGs (*EGFR*, *SOCS2* and *GHR*), the present GBM series (Figure 1C), as well as five independent cohorts accounting for a total of more than 650 additional cases (Figure S2),^9^, ^13^, ^18^, ^19^, ^20^ could be classified into two main groups exhibiting mirror images: (1) GBMs with low‐*GHR* expression and high‐*EGFR*/*SOCS2* expression (*GHR* ^low^/*EGFR* ^high^/*SOCS2* ^high^), and (2) GBMs with high‐*GHR* expression and low‐*EGFR*/*SOCS2* expression (*GHR* ^high^/*EGFR* ^low^/*SOCS2* ^low^). Coherently, in these series, the expression of GHR versus EGFR or SOCS2 was significantly inversely correlated (*p* < .0001).

The GHR is the archetype of the cytokine receptor family, which includes non‐tyrosine kinase receptors that signal through various associated kinases.^21^ Growth hormone (GH) binding to predimerized GHR induces conformational changes leading to the phosphorylation of GHR‐bound JAK2 tyrosine kinase that, in turn, phosphorylates downstream effectors, including the GHR and STATs, resulting in the activation of the JAK‐STAT pathway.^22^ *SOCS2* is a typical target gene of the canonical GHR/JAK2/STAT5b pathway.^23^, ^24^ SOCS2 protein is the main negative regulator of GHR signalling^25^, ^26^, ^27^ acting at two levels: interference with STAT5 recruitment to the GHR complex, leading to interruption of GHR‐induced JAK/STAT signalling, and downregulation of GHR protein through direct ubiquitination and proteasome‐mediated degradation.^27^

Although *SOCS2* expression is a marker of GHR activity, the inverse correlation between *GHR* and *SOCS2* expression in our GBM cohorts was thus intriguing and led us to explore the functionality and role of GHR signalling pathway in GBM. To investigate further the impact of high‐*GHR* expression in GBMs, a subgroup termed GBM^GHR high^ was determined in our cohort on the basis of GHR mRNA expression measured by RT‐qPCR (Figure 1D). GBM^GHR high^ represented 33.3% of our cohort. The differential *GHR* expression by RT‐qPCR was highly significant (fourfold increased expression in GHR^high^ vs. GBM^GHR low^ cases, *p* < .0001) (Figure 1D). As determined by immunohistochemistry analysis using an anti‐GHR antibody directed against the intracellular domain,^28^ GHR protein expression was detected in at least 30% of tumour area of GHR^high^ cases (Figure 1E). Phosphorylated (p)‐STAT5, a canonical target of GHR signalling, was detected in the nucleus of many cells within positive GHR staining regions (Figure 1E). Importantly, the analysis of Ivy Glioblastoma Atlas project dataset showed that GHR mRNA expression was significantly enriched in the infiltrating tumour (IT) anatomical structure compared to the core of the tumour, termed here cellular tumour (CT) (Figure 1F).

Comparison between patients with GBM^GHR high^ or GBM^GHR low^ from ONT series revealed no difference in terms of prognosis (14.47 months vs. 14.00 months, *p* = .87), age at diagnosis and sex ratio (Figure S1B–D). GBM^GHR low^ samples being characterized by high EGFR expression (Figure 1C), this overall suggests that the clinical impact of GHR overexpression was comparable to that of the oncogenic driver *EGFR*. The clinical and molecular significance of GHR was also assessed in TCGA dataset.^13^, ^29^ In this series, patient prognosis was not impacted either by GHR expression status. Of note, no amplification, deletion or somatic mutation of *GHR* were reported in this GBM series. Expectedly, *EGFR* amplified or mutant cases were underrepresented in GBM^GHR high^ (Figure 1G). Distribution analysis with previously described molecular and epigenetic subtypes^12^, ^30^ revealed that the molecular subtypes were distributed differently between GBM^GHR high^ and GBM^GHR low^ (Figure 1G). Indeed, an enrichment of glioma CpG island methylator phenotype (G‐CIMP) and mesenchymal subtypes was observed in TCGA GBM^GHR high^ samples, whereas the classical and proneural subtypes were underrepresented (Figure 1G). *Isocitrate dehydrogenase 1* (*IDH1*) somatic mutant R132H cases, which are associated with G‐CIMP subtype,^30^ were found to be enriched in GBM^GHR high^ from ONT (18% in GBM^GHR high^ vs. 0% in GBM^GHR low^ cases) and TCGA cohorts (14% in GBM^GHR high^ vs. 2% in GBM^GHR low^ cases) (Figure S1E), albeit constituting a minority of GBM^GHR high^ cases in these series.

Together, these analyses identified GHR signalling as a novel candidate oncogenic pathway that defines a distinct molecular subset of GBMs devoid of canonical *EGFR* overexpression, and presenting prognoses comparable to *EGFR*‐driven GBMs.

### 2.2 GHR signalling is active in preclinical models of GBM^GHR high^

**FIGURE 2:**
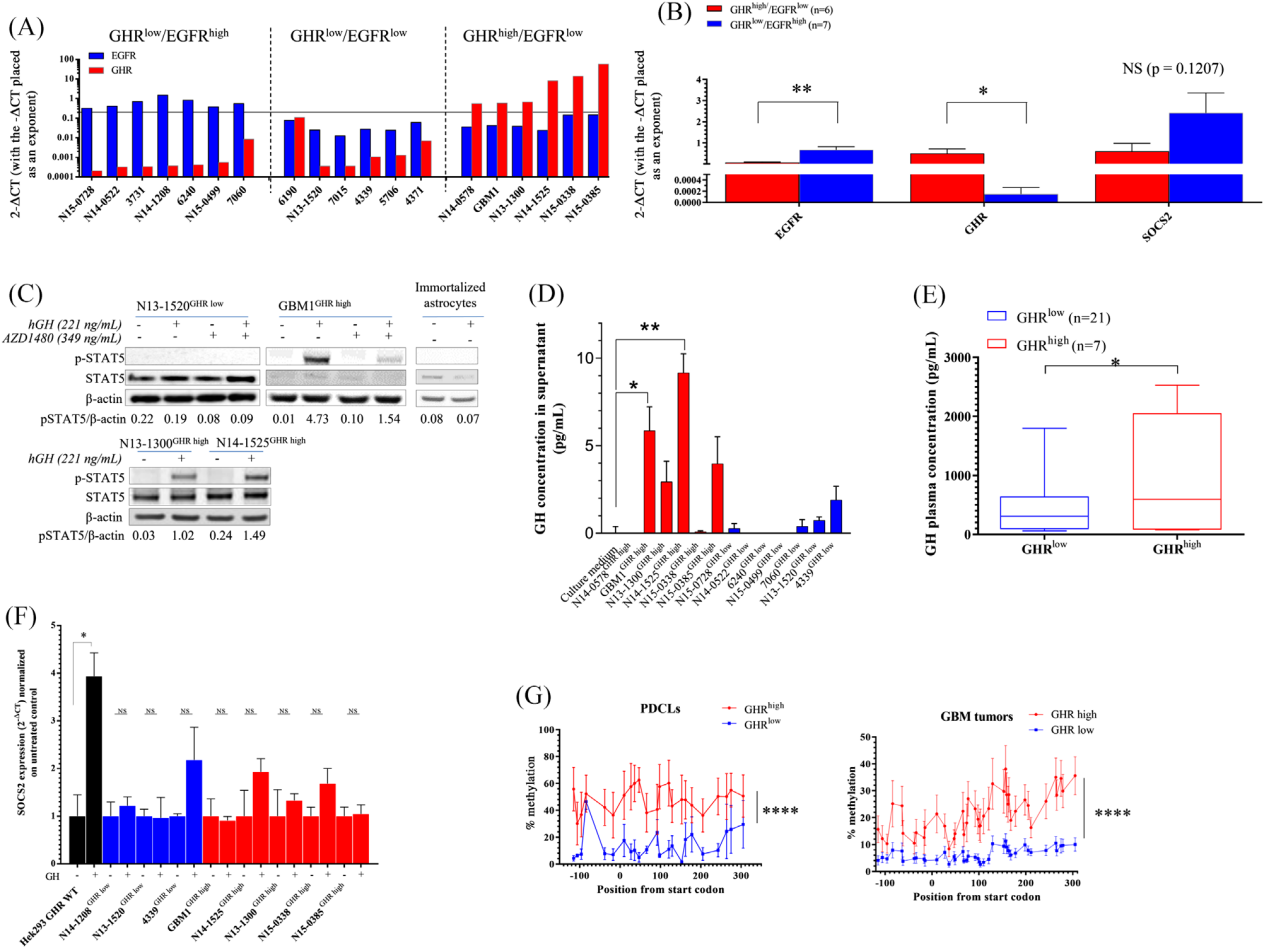
Growth hormone receptor (*GHR*) overexpression is linked with functional STAT5 signalling in vitro, increased circulating hGH in patients and increased suppressor of cytokine signalling 2 (*SOCS2*) promoter methylation. (A) *GHR* and epidermal growth factor receptor (*EGFR*) expression levels in a series of 19 glioblastoma (GBM) patient‐derived cell lines (PDCLs) as determined by RT‐qPCR. *Y*‐axis represents 2^−Δ^ *^CT^* for each gene relative to *PPIA* expression. The threshold of 2^−Δ^ *^CT^* = .2 (80th percentile value for both genes) is represented by the grey line. (B) Expression data of *GHR*, *EGFR* and *SOCS2* of samples shown in panel A were averaged for GHR^low^ and GHR^high^ subgroups. (C) Western blot showing p‐STAT5 in PDCLs exposed to 221‐ng/ml growth hormone (GH) (10 nM) with or without 349‐ng/ml AZD1480 JAK2 inhibitor (1 μM). Densitometric values were normalized to actin levels. (D) Concentration of GH (pg/ml) detected in supernatants of GBM PDCLs after 45 h of culture. **p ≤ .01; *p ≤ .05 compared to culture medium. (E) Plasmatic GH concentration in patients with GBM^GHR high^ or GBM^GHR low^ (*n* = 28). (F) *SOCS2* expression level in PDCLs from GHR^low^ and GHR^high^ groups exposed (+) or not (−) to 221 ng/ml (10 nM) GH for 24 h. HEK293 cells stably overexpressing GHR (HEK293 GHR wild type [WT]) were used as positive control. (G) *SOCS2* promoter methylation in a panel of PDCLs (*n* = 9) or GBMs tumours (*n* = 20), grouped as GHR^low^ or GHR^high^. ****p ≤ .0001 as calculated by ANOVA two‐way test

To assess further the relevance of GHR pathway in GBM^GHR high^, we first quantified *GHR* and *EGFR* expression by RT‐qPCR in a series of 19 GBM patient‐derived cell lines (PDCLs) cultivated in neural stem cell conditions (deprived of serum). We used a threshold of 2^−Δ^ *^CT^* = .2 (80th‐percentile) to identify GBM PDCLs with high levels of *GHR* or *EGFR* expression. Although 6/19 PDCLs exhibited EGFR and GHR mRNA levels below this stringent threshold (Figure 2A), *GHR* and *EGFR* overexpression was identified in 6/32 (18.8%) and 7/32 (21.9%) GBM PDCLs, respectively (Figure 2A). Remarkably, *GHR* and *EGFR* overexpression were mutually exclusive. On average, the *GHR* expression level was dramatically elevated in GHR^high^ versus GHR^low^ PDCLs, and the latter displayed significantly higher *EGFR* expression level than the former (Figure 2B), consistent with what was observed in GBM tissue samples. In these basal culture conditions (i.e. deprived of exogenous GH), *SOCS2* expression levels were not different between both PDCL groups.

To investigate GHR function in GBM PDCLs, GBM1^GHR high^, N13‐1300^GHR high^, N14‐1525^GHR high^ and N13‐1520^GHR low^ were exposed to human GH for 15 min. STAT5 phosphorylation was used as a marker of JAK/STAT signalling activation and assessed by immunoblotting (Figure 2C). The level of p‐STAT5 was increased in the three GBM^GHR high^ cell lines, whereas no change could be detected in N13‐1520^GHR low^. As expected, the addition of JAK2 inhibitor AZD1480 reduced GH‐induced p‐STAT5 in GBM1^GHR high^. Exposure of human immortalized astrocytes to GH did not activate STAT5.

Next, we addressed whether GBM PDCLs expressed GH by measuring the levels of secreted hormone in culture supernatants using ELISA (Figure 2D). No GH was detected in neurosphere culture supernatants used as controls. Globally, GH expression was higher in GHR^high^ (red bars) than in GHR^low^ (blue bars) GBM cells. Two out of the six PDCLs^GHR high^ tested produced significant amounts of GH (N14‐1525 and GBM1), and two PDCLs^GHR high^ produced detectable amounts of GH yet not statistically different from values measured in the supernatant (*p* = .174 and .161, for N13‐1300 and N15‐0385, respectively). Such levels (sub‐pM range) were nevertheless too low to induce detectable GHR signalling in routine immunoblotting conditions (Figure 2C, control untreated conditions). None of the seven PDCLs^GHR low^ tested produced detectable levels of GH. Interestingly, although GH plasma level in GBM patients fell within the normal range of healthy adults (200–2000 pg/ml),^31^ GBM^GHR high^ patients exhibited ∼2‐fold higher GH plasma levels than GBM^GHR low^ patients (mean of 419 pg/ml in GBM^GHR low^ patients vs. 1026 pg/ml in GBM^GHR high^ patients; Figure 2E).

Together, these data indicate that PDCL^GHR high^ are more responsive to GH than their GHR^low^ counterparts. In patients, GBM^GHR high^ is associated with increased levels of circulating GH.

### 2.3 GBM^GHR high^ is linked to a disruption of SOCS2‐dependent negative regulation through SOCS2 promoter hypermethylation

We then aimed to elucidate the mechanism underlying low‐*SOCS2* expression in GBM^GHR high^ (Figure 1C). SOCS2 participates in the downregulation of GHR protein through direct ubiquitination and proteasome‐mediated degradation and is normally upregulated upon GHR signalling activation.^27^ We thus hypothesized that this negative feedback mechanism may no longer be functional in GBM^GHR high^, thus preventing GHR downregulation and subsequent inhibition of GHR signalling. To address this hypothesis, we analysed the ability of GH to induce *SOCS2* expression in various PDCLs, using GHR‐expressing HEK293 cells as a positive control. In non‐stimulated PDCLs, the basal levels of *SOCS2* were comparable between GHR^high^ and GHR^low^ cells (Figure 2B). In agreement with undetectable GHR/STAT5 signalling in GHR^low^ PDCLs exposed to GH, no significant GH‐mediated upregulation of *SOCS2* could be observed in three PDCL^GHR low^ (Figure 2F). The trend observed for 4339 may reflect a ∼3‐fold higher level of GHR expression compared to the N14‐1208 and N13‐1520 (Figure 2A). In‐line with our hypothesis, GH failed to induce *SOCS2* expression in five PDCL^GHR high^ (Figure 2F) despite dramatically higher levels of GHR expression (Figure 2A) and marked STAT5 phosphorylation (Figure 2C). Hypermethylation of *SOCS2* promoter was previously reported as a possible mechanism preventing *SOCS2* upregulation.^32^ We thus investigated *SOCS2* promoter DNA methylation data in a panel of 9 PDCLs and 20 GBM tissue samples (Figure 2G). The data show a clear increase in *SOCS2* promoter methylation in PDCLs^GHR high^ and GBM^GHR high^ compared to their GHR^low^ counterparts (Figure 2G).

Overall, our data indicate that the disruption in GH‐mediated upregulation of *SOCS2* expression in GBM^GHR high^ is linked with *SOCS2* promoter methylation in this subgroup.

### 2.4 Activation of GHR signalling in GBM modulates cell migration and invasion

To decipher the consequences of GHR overexpression in GBM cells while avoiding the interference of confounding factors due to the natural history and molecular context specific to each GBM from which GHR^low^ and GHR^high^ PDCLs were generated, *GHR*‐overexpressing PDCLs were generated by stable transduction of GHR^low^ PDCLs (4339 and N13‐1520) with expression vectors encoding human wild type (WT)‐GHR or a constitutively activated (CA) form of rabbit GHR (CA‐GHR).^33^, ^34^ This CA‐GHR vector encodes an engineered form of GHR in which the extracellular domain has been replaced by leucine‐zipper sequences, which ensures the formation of GH‐insensitive GHR dimers exhibiting constitutive signalling activity.^33^ A GFP‐encoding vector was used as a control. Validation of these transformed PDCLs is shown in Figure S4A–D. As expected,^33^, ^34^ mild constitutive STAT5 signalling was observed only in 4339^CA‐GHR^ or N13‐1520^CA‐GHR^. GH stimulation markedly upregulated STAT5 signalling GH in 4339^WT‐GHR^ and N13‐1520^WT‐GHR^, but not in 4339^CA‐GHR^ or N13‐1520^CA‐GHR^, in agreement with the insensitivity of rabbit CA‐GHR construct to human GH.

**FIGURE 3:**
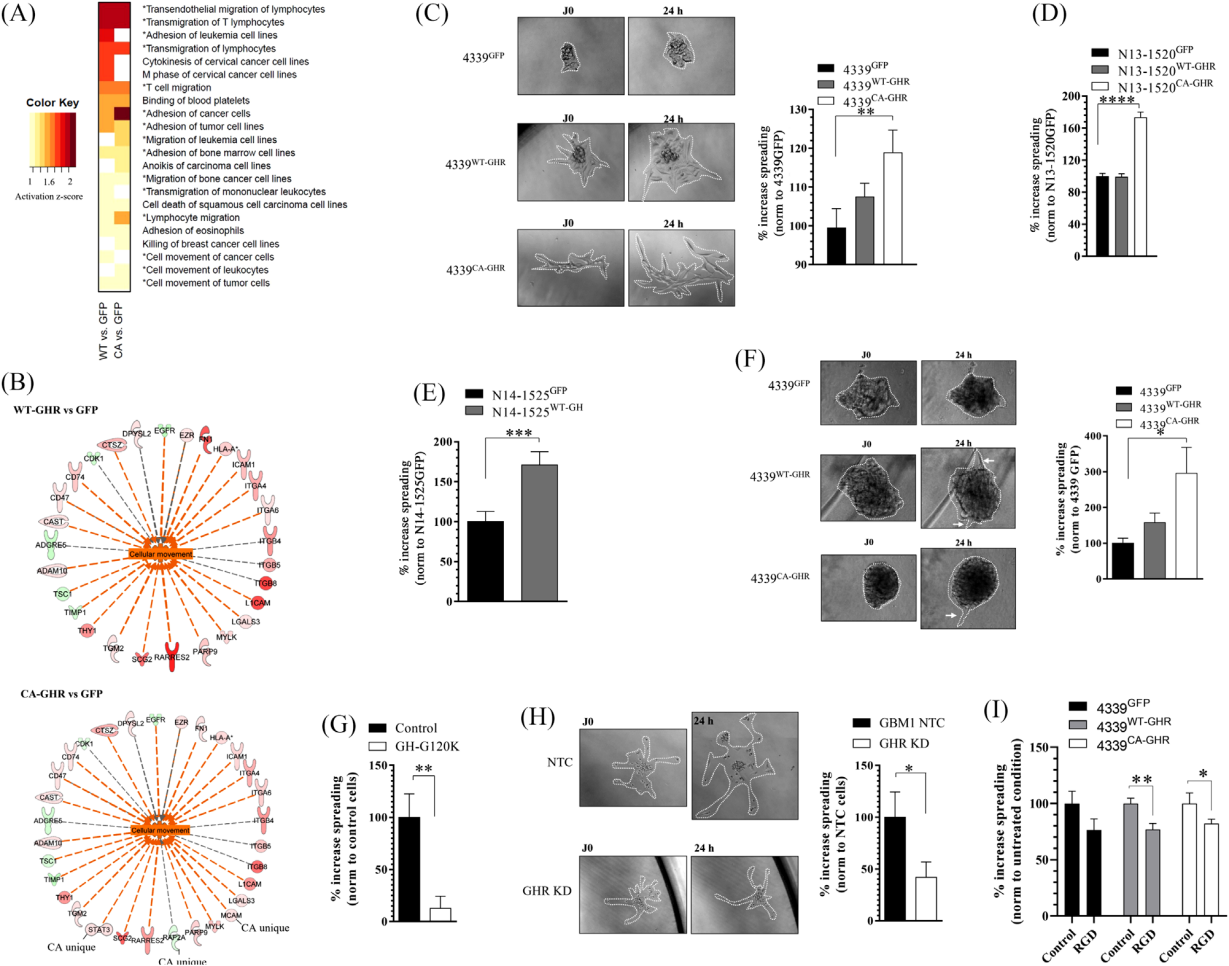
Activation of growth hormone receptor (GHR) signalling promotes cell migration and invasion in vitro. (A) Most activated biological functions in 4339^WT‐GHR^ or 4339^CA‐GHR^ versus 4339^GFP^ (*p* ≤ .005) inferred from global proteomic analysis. Functions associated with cellular movement are marked with an asterix. (B) Network of molecules whose expression status (up/shades of red, or down/shades of green, *p* < .05) is predicted to promote activation of cell movement in 4339^WT‐GHR^ versus 4339^GFP^ or 4339^CA‐GHR^ versus 4339^GFP^ (activation *z*‐score +.9). (C–E) In vitro cell migration assays of patient‐derived cell lines (PDCLs) 4339^WT‐GHR^ or 4339^CA‐GHR^ versus 4339^GFP^ with representative micrographs (C), N13‐1520^WT‐GHR^ or N13‐1520^CA‐GHR^ versus N13‐1520^GFP^ (D) and N14‐1525^WT‐GH^ versus N14‐1524^GFP^ (E). *Y*‐axis represents the percentage of increase in sphere area over 24 h. (F) In vitro cell invasion assays of 4339^WT‐GHR^ or 4339^CA‐GHR^ versus 4339^GFP^ and representative micrographs. *Y*‐axis represents the percentage of increase in sphere area over 24 h. (G) Effect of GHR signalling inhibition on in vitro migration of GBM1^GHR high^. Cells were exposed to 20‐μg/ml hGH‐G120K for 24 h. (H) Effect of GHR expression inhibition on in vitro migration of GBM1^GHR high^. CRISPR‐induced GHR knockdown (KD) is compared to non‐targeted control (NTC). (I) Effect of RGD peptide integrin antagonist on in vitro migration of 4339^GFP^, 4339^WT‐GHR^ and 4339^CA‐GHR^. Cells were exposed to 1‐μg/ml RGD for 24 h. *p ≤ .05; **p ≤ .01; ***p ≤ .001; ****p ≤ .0001

The analysis of the global proteome of each cell population was then undertaken. Raw data have been deposited to the ProteomeXchange Consortium via the PRIDE^35^ partner repository (identifier PXD004969). Proteomic expression data from 4339^WT‐GHR^ (in the absence of hGH) or 4339^CA‐GHR^ were compared to the data from 4339^GFP^ (Curie proteomic series, *n* = 3). Compared to 4339^GFP^, we found 929 and 951 proteins differentially expressed (*p* < .05, log2 fold change ≥ |1|) in 4339^WT‐GHR^ and 4339^CA‐GHR^ cell lines, respectively, with 728 proteins common to both lists. The most differentially expressed proteins are listed in Tables S3 and S4 and compared in Figure S5. IPA of differentially expressed proteins revealed that, among the functions with the highest predicted activation *z*‐score for WT‐GHR and CA‐GHR (activation *z*‐score ≥ .9), >70% of listed functions (13/18 and 12/17, respectively) were associated with cellular movement (Figure 3A). The differentially expressed proteins whose expression status (up‐ or downregulation) is consistent with the activation of these biological processes are displayed in Figure 3B. They include several members of the integrin family, including integrin α4, α6, β4, β5 and β8. An independent proteomic acquisition was undertaken that validated this observation and confirmed the increased expression of integrin α6 and β4 in 4339^WT‐GHR^ and 4339^CA‐GHR^ compared to 4339^GFP^.

In vitro functional assays using two transduced PDCLs (4339 and N13‐1520) expressing WT‐ or CA‐GHR constructs were then conducted to assess the impact of GHR expression on GBM cell movements involved in cell migration and invasion. All assays were performed in the absence of GH addition. To assess 2D in vitro migration, we quantified the spreading of GBM neurospheres deposited on laminin‐coated culture plates. After 24 h, 4339^CA‐GHR^ cells exhibited significantly increased migration capacity (Figure 3C) compared to 4339^GFP^ cells. Similarly, N13‐1520^CA‐GHR^ exhibited increased migration activity compared to N13‐1520^GFP^ (Figure 3D). The ectopic addition of GH to the assay did not result in an increased migration of WT‐GHR transduced cells (not shown), suggesting that the effect of exogenous GH is too transient^36^ to translate into a significant phenotype.

Next, to assess the impact of a constant GH stimulation on cell migration, we transduced the naturally GHR‐overexpressing N14‐1525^GHR high^ PDCL with GH (vs. GFP) expression vectors (Figure S4E,F). Coherently, an increase in migration was observed when N14‐1525^GHR high^ overexpresses GH (Figure 3E).

In vitro invasiveness was assessed by depositing neurospheres in Cultrex Basement Membrane Extract (BME). 4339^CA‐GHR^ was found to exhibit significantly increased invasion capacity (Figure 3F) compared to 4339^GFP^, as demonstrated by the presence of cellular extensions (white arrows) in the BME matrix.

The impact of GHR inhibition in GBM cell line was then assessed in vitro using both a pharmacological approach (GH‐G120K competitive antagonist) and a genetic knockdown (KD) using the CRISPR/Cas9 gene‐editing technology. *GHR* KD was achieved by inducing a heterozygous deletion of *GHR* exon 3 in GBM1^GHR high^ cell line (Figure S4G), which was confirmed by Sanger sequencing. Both GH‐G120K and *GHR* KD markedly reduced 2D in vitro cell migration of GBM1^GHR high^ (Figure 3G,H).

Finally, to confirm the role of the integrins in GHR‐driven cell migration, the impact of an RGD peptide, a broad integrin antagonist^37^ reported to block cell attachment to the extracellular matrix and inhibit cell migration,^38^ was assessed on 4339^GFP^, 4339^WT‐GHR^ and 4339^CA‐GHR^ cells. RGD significantly decreased cell migration in 4339^WT‐GHR^ and 4339^CA‐GHR^ (Figure 3I), whereas no significant effect was observed on 4339^GFP^.

Taken together, these data suggest that the activation of *GHR* signalling in GBM is associated, in vitro, with increased (1) expression of proteins involved in cellular movement, in particular members of the integrin family; (2) cell migration and (3) cell invasion. Conversely, the inhibition of GHR expression and signalling reduces cell migration.

### 2.5 Activation of GHR signalling activates cell proliferation

**FIGURE 4:**
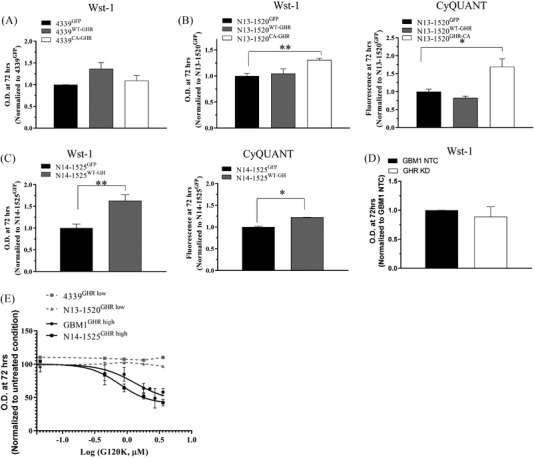
Activation of growth hormone receptor (GHR) signalling increases proliferation in vitro. (A–C) In vitro cell proliferation of 4339^WT‐GHR^ and 4339^CA‐GHR^ versus 4339^GFP^ (A), N13‐1520^WT‐GHR^ and N13‐1520^CA‐GHR^ versus N13‐1520^GFP^ (B), N14‐1525^WT‐GH^ versus N14‐1524^GFP^ (C) and GBM1 NTC versus GHR KD (D) was measured using Wst‐1 assay and, if significant, validated using CyQUANT assay, as indicated. *Y*‐axis represents optical density or fluorescence at 72 h relative to GFP condition. *p ≤ .05; **p ≤ .01. (E) In vitro cell proliferation (Wst‐1) of GHR^high^ and GHR^low^ cell lines in response to GH‐G120K. *Y*‐axis represents optical density at 72 h relative to untreated cells in response to GH‐G120K concentrations shown as Log [μM].

We then evaluated the impact of GHR/GH constructs on cell proliferation in vitro. Although no statistical difference was observed for 4339^WT‐GHR^ and 4339^CA‐GHR^ compared to 4339^GFP^ (Figure 4A), cell proliferation was increased in N13‐1520^CA‐GHR^ compared to N13‐1520^GFP^ as demonstrated by two different viability assays (wst‐1 and CyQUANT; Figure 4B). Consistently, the overexpression of GH in N14‐1525^GHR high^ (N14‐1525^WT‐GH^) promoted cell proliferation compared to N14‐1525^GFP^ as demonstrated using the same two assays (Figure 4C). However, GHR KD did not significantly impact cell proliferation in GBM1^GHR high^ cell line (Figure 4D), even though the impact on migration was clear (Figure 3H), suggesting a more prominent role of GHR on cell migration in this model. Finally, exposure to GH‐G120K for 96 h decreased the cell viability of GHR^high^ (GBM1^GHR high^ and N14‐1525^GHR high^) but not of GHR^low^ (N13‐1300^GHR low^ and N13‐1520^GHR low^) PDCLs (Figure 4E).

Overall, the activation of GHR signalling increases cell proliferation in GBM, an effect that can be prevented by pharmacological GHR inhibition in GHR^high^ PDCL but not in GHR^low^ PDCLs.

### 2.6 Activation of GHR signalling supports tumourigenicity

**FIGURE 5:**
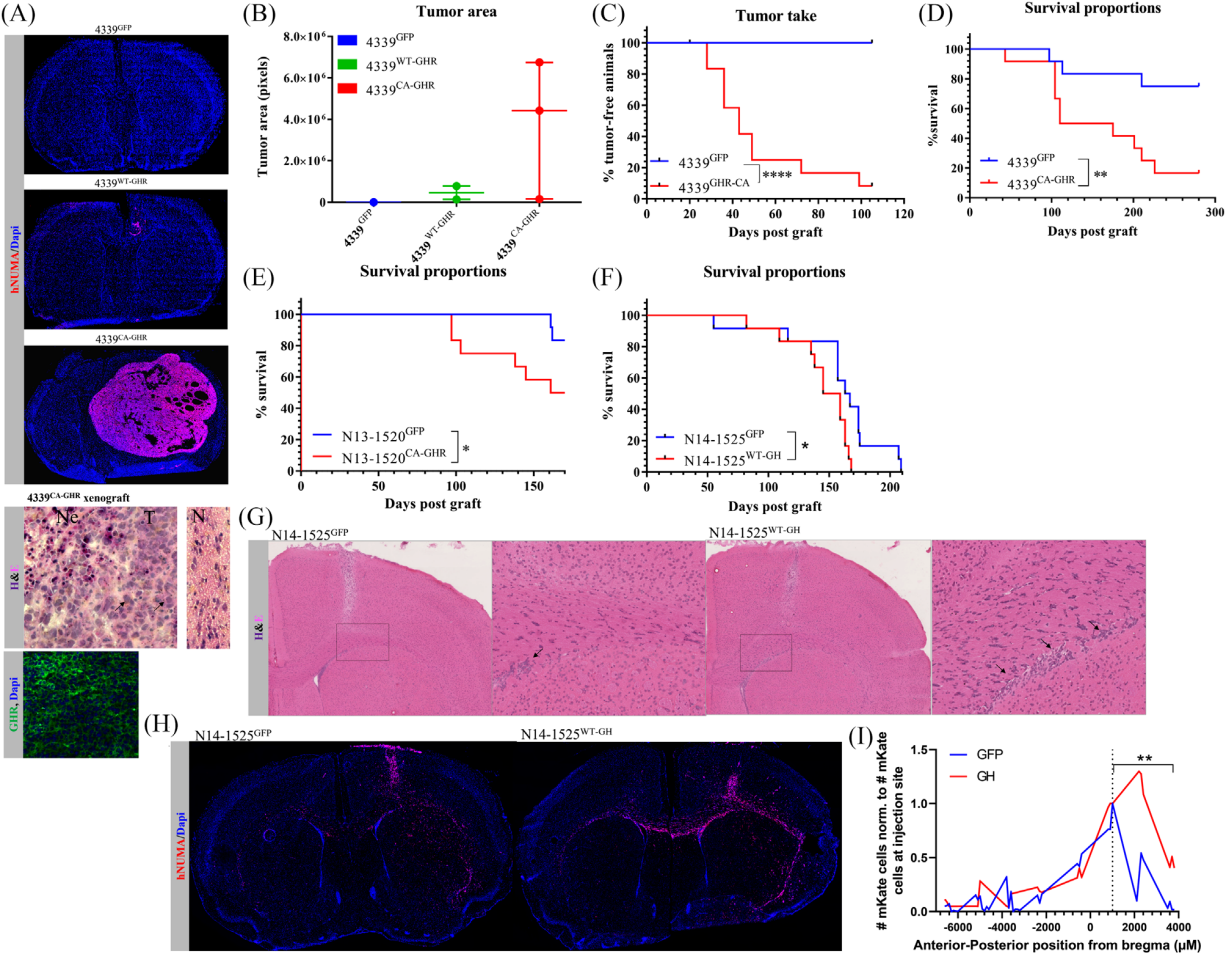
Growth hormone receptor (GHR) overexpression or GHR signalling activation promotes tumourigenesis and tumour growth. (A) Immunofluorescence micrographs of mouse brains 5 months after inoculation of 4339^WT‐GHR^, 4339^CA‐GHR^ and 4339^GFP^ showing human‐specific nuclear mitotic antigen (hNuMA) (red), DAPI (blue) and GHR (green). H&E‐stained tissue sections are shown such as normal brain tissue (N), tumour (T), necrosis (Ne) and mitotic events (arrows). (B) Tumour area quantified in pixels from hNuMA‐stained brain sections. (C) Percentage of mice showing established tumours over time following an inoculation of 4339^CA‐GHR^ and 4339^GFP^. ****p ≤ .001. (D) Survival of mice following an inoculation of 4339^CA‐GHR^ and 4339^GFP^. **p ≤ .01. (E) Survival of mice following inoculation of N13‐1520^CA‐GHR^ and N13‐1520^GFP^. *p ≤ .05. (F) Survival of mice following inoculation of N14‐1525^WT‐GH^ and N14‐1525^GFP^. *p ≤ .05. (G) H&E‐stained tissue of mouse brains 3.5 months after the inoculation of N14‐1525^WT‐GH^ and N14‐1525^GFP^ showing clusters of invasive glioblastoma (GBM) cells in the corpus callosum (purple nuclei shown by the arrows). (H) Immunofluorescence micrographs of mouse brains 3.5 months after the inoculation of N14‐1525^WT‐GH^ and N14‐1525^GFP^ showing human GBM cells (detected by human‐specific nuclear mitotic antigen – hNuMA – in red) and DAPI (blue). (I) Quantification of hNuMA‐positive cells in coronal sections of entire brains harvested from N14‐1525^WT‐GH^ and N14‐1525^GFP^ grafted mice (3.5 months post cell inoculation) normalized to the number of hNuMA‐positive cells at the inoculation site (Bregma + 1000 μM). **p ≤ .01

We then analysed the tumour‐initiating capacity of the three transduced 4339 PDCLs after intra‐striatal inoculation in immunocompromised Nude mice. Five months after surgery, both 4339^WT‐GHR^ (2/3 mice) and 4339^CA‐GHR^ (3/3) cell lines generated tumours in mouse brain, whereas 4339^GFP^ did not (0/3) (Figure 5A). On average, CA‐GHR‐generated tumours were eight times bigger than WT‐GHR‐generated tumours, as evaluated by area measurement in hNuMA‐stained brain sections (Figure 5B). Histopathological analysis of tumours revealed a proliferation of poorly differentiated astrocytic cells and of undifferentiated cells with a high nucleocytoplasmic ratio, numerous mitosis and areas with necrosis, suggestive of GBM tumours with a primitive component (Figure 5A). This aspect was reminiscent of the parental GBM from which 4339 PDCL was derived (Figure S6). These results were validated in a second study in which 4339^GFP^ and 4339^CA‐GHR^ were transduced to express the luciferase gene then grafted into the striatum of mice (12 mice/group). The time post‐graft when tumours were considered established (10‐fold increase in bioluminescence signal) (Figure 5C) showed that although 4339^CA‐GHR^‐generated tumours were established at a median of 43‐day post‐grafting, no mice grafted with 4339^GFP^ reached this threshold. Mouse survival was also significantly impacted by CA‐GHR expression (Figure 5D). N13‐1520^CA‐GHR^ and N13‐1520^GFP^ were also grafted into mouse brain (12 mice/group). In this model, the CA‐GHR construct did not impact the time to tumour establishment (not shown) but significantly impacted mouse survival (Figure 5E).

Next, N14‐1525^WT‐GH^ and N14‐1525^GFP^ were grafted into the striatum of mice. Although GH expression did not impact the time to tumour establishment (not shown), it significantly reduced mouse survival (Figure 5F). Histopathological analysis of tumours revealed in both conditions the presence of highly invasive GBM cells in locations remote from the injection site, that is in the corpus callosum of both hemispheres, albeit a greater number of these invasive cells was observed in the GH condition (Figure 5G,H). Quantification of hNuMA‐positive cells throughout entire brains from these mice confirmed an increased invasion of N14‐1525^WT‐GH^ cells from the injection site towards the anterior regions (corpus callosum and striatum) of the brain (Figure 5I).

Taken together, these results strongly support that GHR signalling promotes tumourigenicity, proliferation and invasiveness of GBM cells.

## 3 DISCUSSION

We here identified GHR overexpression and active GHR downstream signalling as a novel oncogenic pathway in a subset of GBM (GBM^GHR high^) falling out of the well‐characterized subgroup of *EGFR*‐overexpressing GBMs. *SOCS2*, the major negative regulator of GHR pathway,^25^, ^26^, ^27^ is downregulated in GBM^GHR high^, presumably contributing to the maintenance of high‐GHR expression and signalling. The fact that two genes acting in the same intracellular signalling pathway are modulated in a consistent direction (Figure 1) strongly supports the importance of the GHR pathway in this GBM subgroup, representing up to 30% of cases. The mutually exclusive *GHR* ^high^/*SOCS2* ^low^/*EGFR* ^low^ and *GHR* ^low^/*SOCS2* ^high^/*EGFR* ^high^ expression patterns identified in the present series were also observed in five additional independent datasets. As both EGFR and GHR are able to activate common signalling pathways (JAK2, STATs, Src, and MAPK),^39^, ^40^ we here propose that, in low EGFR‐expressing GBMs, GHR overexpression can take over and activate key signalling pathways driving oncogenesis.

In GBM, enhanced cell invasion is a major obstacle for efficient surgical resection, tumour cells dispersed in the parenchyma being a primary source of tumour relapse.^41^ Here, we suggest GHR signalling to be critical for cell migration and invasion in GBM. We found that the proportion of GBM from the mesenchymal transcriptional subtype, which is associated with more invasive features than the other subtypes,^41^ was overrepresented in GBM^GHR high^. Coherently, EMT is predicted to be modulated in our low‐*EGFR*‐expressing GBM cohort (Figure 1A). Moreover, we report that GHR mRNA expression is enriched in the infiltrative tumour of patient GBM compared to the core of the tumour (Figure 1F). In addition, we found that cell movement appears to be one of the main biological functions activated by *GHR* in our GBM models. Indeed, in our GBM PDCLs, GHR signalling activates cell migration and invasion in vitro (Figure 3). In vivo, we show that a continuous activation GH/GHR signalling in GBM cells promotes increased cell invasion throughout mice brains (Figure 5I). Our study shows that *GHR* overexpression modulates the expression of multiples genes and related proteins involved in cellular movement, including members of the Integrin family. In particular, integrins α6 and β4 form a complex laminin receptor that was shown to be an important regulator of tumour invasion.^42^, ^43^ The reduction of GHR‐dependent GBM cell migration in the presence of RGD peptide confirmed the relevance of integrin as contributors to this effect (Figure 3I). Metalloproteinase ADAM10 was also found to be upregulated in *GHR*‐overexpressing PDCLs, and, coordinately, the metalloproteinase inhibitor TIMP1 was downregulated (Figure 3B). These observations are consistent with a previous report showing that, in mammary carcinoma, GHR signalling promotes the expression of fibronectin and matrix metalloproteinase activity in association with epithelial‐to‐mesenchymal transition (EMT).^44^ Overall, our data support a strong link between GHR and the regulation of cell migration and invasion in vitro, in vivo and in patient tumours.

Data from the literature converge towards the oncogenic role of the GHR axis: GHR activates oncogenic signalling pathways, including pathways promoting EMT, cell survival, migration/invasion and resistance to therapy (reviewed in Refs. [^22^, ^45^]). GHR signals mainly through JAK2 and Src family kinases, leading to the activation of STAT, MAPK and PI3K pathways,^22^ as well as the modulation of long non‐coding RNAs, as reported recently.^46^ However, the consequences of *GHR* overexpression in GBM have remained largely unstudied. Our study reports the pro‐tumoural consequences of GHR overexpression and/or activation in GBM, including in vitro cell proliferation, in vivo tumour take rate, survival and invasiveness in GBM‐bearing animals, which suggests the involvement of GHR in a large spectrum of biological functions associated with GBM progression. Consistent with our data, high levels of GH and GHR expression were also reported by others in glioma cell lines compared to normal brain tissue,^47^, ^48^ further supporting the oncogenic role of GHR signalling in GBM. In our PDCL^GHR high^, we showed that GHR signalling activates the canonical STAT5 pathway. Whether GHR signalling also acts through other pathways in *GHR*‐overexpressing GBM remains to be evaluated.

*GHR* overexpression is frequently observed in tumours.^22^, ^49^, ^50^, ^51^, ^52^, ^53^ In breast cancer, as in GBMs, *GHR* expression is quite heterogeneous and 12% of cases overexpress *GHR* (239 out of 1980, mRNA *z*‐score 1.3^54^, ^55^). In rectal cancer, *GHR* overexpression was associated with poor response to radiotherapy.^56^ The mechanisms involved in GHR overexpression are at present unknown, including in GBMs.^57^ In this respect, our observation that *SOCS2* is downregulated in GBM^GHR high^ was particularly interesting, as SOCS2, in addition to act as a negative JAK/STAT signalling regulator, was also shown to regulate cellular GHR levels through direct ubiquitination and proteasome‐mediated degradation, and accordingly, *SOCS2* knockout resulted in increased GHR protein expression.^27^ Our observation is intriguing, however, because *SOCS2* expression is normally activated by GHR through STAT5 signalling as a negative feedback regulation mechanism,^23^ and we showed that STAT5 signalling is functional in PDCLs^GHR high^. Importantly, we found that the methylation of *SOCS2* promoter is increased in both GHR^high^ PDCLs and GBMs. Methylation of *SOCS2* promoter was previously reported as a possible mechanism of SOCS2 downregulation^32^ and described in GBM.^58^ Together, our data suggest that, in agreement with CA STAT signalling identified using IPA, the GHR pathway may be upregulated in GBM^GHR high^ tumours at several levels: *GHR* transcription (GHR^high^ vs. GHR^low^ PDCL), GHR protein stabilization and GHR signalling (SOCS2 downregulation).

Our in vitro data demonstrate that GHR overexpression increases GBM cell responsiveness to GH. In patients, different sources of GH may contribute to trigger GHR signalling in GHR^high^ GBM cells: (1) GHR^high^ GBM cells themselves (autocrine GH), (2) neighbouring cells (paracrine GH) and (3) circulating GH secreted by the pituitary gland (endocrine GH). In our GBM cohort, *GHR* expression levels in bulk tumours and GH plasma levels were correlated, suggesting a possible contribution of circulating GH to tumour progression in these patients. Indeed, *GHR*‐overexpressing cells may be positively enriched in an environment where GH levels are abundant. The capacity of circulating GH to reach the brain and induce signalling in cells from the central nervous system is well described.^59^ In mice, the injection of exogenous GH leads to a rapid accumulation of the hormone in the parenchyma of the brain.^60^ The contribution of locally produced GH is also possible. Indeed, we showed that a proportion of GBM PDCLs secrete GH (Figure 2D), as frequently observed in cancer.^48^, ^61^ The steady‐state level of constitutive signalling induced by locally produced hormones is usually much lower, hence more difficult to detect, than signalling induced by acute hormonal stimulation. This presumably reflects that sustained GHR signalling has integrated negative regulatory mechanisms intrinsically maintained by signalling per se (e.g. phosphatases, receptor internalization/degradation). Still, this moderate cell‐autonomous GHR signalling induced a series of biological responses of GHR^high^ GBM cells that could be downregulated by pharmacological GHR inhibition (Figure 4E). This is reminiscent of other reports showing that tumour‐secreted GH promotes the invasive phenotype in mammary carcinoma.^44^ Interestingly, GBM^GHR high^ cells expressing autocrine GH (or CA‐GHR) were insensitive to the addition of exogenous GH as reflected by unaltered levels of STAT5 phosphorylation (Figure S4). This suggests that the effects of circulating versus locally produced GH might be mutually exclusive, as supported by a model described by Van den Eijnden et al. in 2007 where GHR bound to autocrine GH is not available to bind endocrine GH at the cell surface.^61^ Overall, it is likely that *GHR* overexpression sensitizes cells to GH stimulation, originating either from the tumour itself or from the circulation, potentially creating a vicious circle in which survival and expansion of *GHR*‐overexpressing cells are promoted.

In‐line with the tumour‐promoting role of GHR demonstrated in this study using several GBM models, targeted inhibition of GHR signalling using the prototypic GH antagonist GH‐G120K impaired GBM cell migration in vitro and decreased cell viability in PDCLs^GHR high^ only. These data are a first step showing the therapeutic potential of pharmacological GHR inhibition in GBM and warrant further experimental investigations assessing the impact of GHR inhibition in in vivo preclinical models of *GHR*‐overexpressing GBM. Commercially available GHR small molecule inhibitors do not currently exist. Pegvisomant is an efficient PEGylated GH core‐based peptide antagonist currently used in the clinics to counteract excess of GHR signalling in acromegalic patients. This compound was shown to be efficient in various tumour types^62^, ^63^, ^64^, ^65^, ^66^ and to potentiate chemotherapy efficacy.^67^ However, its poor passage across the blood–brain barrier^68^ predicts low efficacy for GBM treatment. Other GHR‐targeted compounds currently in clinical development such as antisense oligonucleotides^69^ could constitute a promising approach, although the capacity of such molecules to reach the brain remains to be assessed. According to the potential role of high‐GH circulating levels in GHR^high^ GBMs (see earlier), strategies reducing GH circulating levels could also provide a therapeutic benefit. In‐line with this, GH releasing hormone inhibitors, which decrease pituitary GH secretion, reduced tumour progression of subcutaneous GBM cell line U87 tumours.^70^ Ultimately, efforts to develop new GHR inhibitors specifically adapted to brain tumour treatment will be necessary.

In conclusion, our study identifies a new subset of *GHR*‐overexpressing GBMs and demonstrates a role for GHR signalling in GBM oncogenesis. GHR signalling pathway therefore emerges as oncogenic signalling pathway in low‐*EGFR*‐expressing GBMs and as a promising therapeutic target.

## 4 MATERIALS AND METHODS

### 4.1 Selection of patients

Human GBM tissue samples were selected from OncoNeuroTek (ONT; Paris Brain Institute) tumour tissue bank. Brain tumours fulfilling the following inclusion criteria were selected from our brain tumour database: (1) histologically proven newly diagnosed supratentorial GBM according to the World Health Organization classification system 2016, concordantly reviewed by two neuropathologists,^2^ (2) a clinical history compatible with a newly diagnosed de novo GBM (no prior history of lower grade tumour), (3) available high‐quality tumour RNA and/or frozen tissue allowing further molecular analysis and (4) written consent obtained from the patient for molecular analysis.

### 4.2 RNA extraction and processing of expression array data

Approximately 50 mg of tissues from the initial 54 GBM samples were used to extract total RNA using the RNeasy Lipid Tissue mini kit (Quiagen, CA), following the instructions of the manufacturer. The quality of RNA obtained was checked using a Bioanalyser System (Agilent Technologies, Paolo Alto, CA) using the RNA Nano Chips. Genome U133 Plus 2.0 Expression arrays data processing was done according to the manufacturer recommendations. Normalization was performed using the RMA method.^71^ Clustering analysis and class comparison using a univariate *t*‐test were performed using dChip software (http://biosun1.harvard.edu/complab/dchip/).^72^ A *p*‐value <.005 was used to define differentially expressed genes. In order to compare the gene expression profile of the gliomas with normal brain, we used the gene expression data of five samples of *corpus callosum* (GSM175855, GSM175856, GSM175857, GSM175858, GSM176050) and five samples of cortex (GSM176049, GSM176344, GSM176345, GSM176346, GSM176347), available in the Gene Expression Omnibus repository (GSE7307).

### 4.3 Expression array experiment

GBM tumour RNA was processed for hybridization on the GeneChip Human Genome U133 Plus 2.0 Expression arrays (Affymetrix, CA) which contains over 54 000 probe sets analysing the expression level of more than 47 000 transcripts and variants, including 38 500 well‐characterized human genes.

### 4.4 Molecular subtypes and genomic alterations from additional series

Expression data were extracted from ONT dataset, as well as five additional publicly available datasets,^9^, ^13^, ^18^, ^19^, ^20^ and plotted using pheatmap *R* function. For the analysis of molecular subtypes and genomic alterations in relation with GHR expression, a series of 273 patients was selected from the TCGA dataset^13^ and accessed at www.cbioportal.org.^29^ *GHR* expression data (available for 160 out of 273 patients only), molecular subtype and gene mutational and copy number status for each sample were obtained from Ref. [^13^]. High‐*GHR* expression status was determined using an mRNA threshold of *z*‐score 1.3.

### 4.5 GHR mRNA expression analysis in tumour anatomical structures

GHR RNA sequencing data were extracted from the Ivy Glioblastoma Atlas project dataset,^73^ which comprises a total of 122 RNA samples generated from 10 tumours and sorted in 5 structures (Leading Edge, Infiltrating Tumour, Cellular Tumour, Microvascular Proliferation, and Pseudopalisading Cells Around Necrosis) identified by H&E staining. The Microvascular Proliferation structure was removed from our analysis to focus on the expression of GHR in tumour cells only.

### 4.6 Cell lines

All GBM PDCLs were established by the GlioTex team (Glioblastoma and Experimental Therapeutics) in the Brain and Spine Institute (ICM) laboratory and maintained at 37°C, 5% CO_2_ in neurosphere growth conditions using DMEM/F12 (Gibco, Life Technologies, Saint‐Aubin, France) culture medium supplemented with 1% penicillin/streptomycin, B27 diluted 1:50 (Gibco), EGF (20 ng/ml) and FGF (20 ng/ml) (Preprotech, Neuilly‐sur‐Seine, France). GBM1 cell line is derived from U87 cell line (purchased from ATCC) in our laboratory and is maintained in neurosphere growth conditions to allow comparisons with the other neurosphere‐cultured PDCLs. HEK293 WT‐GHR were generated as previously described^74^ and cultured in DMEM 10% foetal bovine serum (Gibco, Life Technologies, Saint‐Aubin, France). The identity of all cell lines established at the ICM was confirmed by short tandem repeat assay according to manufacturer's instructions (PowerPlex 16, Promega, Charbonnières‐les‐Bains, France and sequencing performed by Genoscreen, Lille, France) and validated within 3 months of their use for the studies presented here. Of note, all PDCLs used in this study are IDH1 WT. Immortalized human astrocytes (transduced with *hTERT* expression vector) were purchased from ABM (Richmond, Canada) and maintained on collagen‐coated flasks (G422, ABM) in Prigrow IV medium (TM004, ABM) supplemented with 1% penicillin/streptomycin and 10% foetal bovine serum (Gibco, Life Technologies, Saint‐Aubin, France).

### 4.7 RT‐qPCR

First Strand cDNA Synthesis Kit (Fisher Scientific, Illkirch, France) from tumours, non‐tumour controls or cell lines was used to generate cDNA. *GHR*, *EGFR* and *SOCS2* gene expression were confirmed using SYBR Green real‐time quantitative polymerase chain reaction (qPCR) analysis (Absolute SYBR Green Rox Mix, Abgene, Paris, France) or LightCycler 480 Probes Master mix and Universal Probe Library probes specific to each genes. The genes and primers are listed in Table 1. House‐keeping genes used were ALAS1 or PPIA. Real‐time qPCR reactions were performed according to the manufacturer's instructions for each system. The 2^−Δ^ *^CT^* method was used to determine the relative expression, where Δ*CT* = *CT*_target gene_ – *CT*_PPIA or ALAS1_. Final results were expressed as a ratio of the 2^−Δ^ *^CT^* value for the studied gene for each sample normalized to that of the reference sample.

**TABLE 1:** Expression real‐time quantitative PCR primers (unless stated, all genes are human genes)

Gene	Primer forward	Primer reverse	No. of UPL probe (if any)
GHR^a^	Qiagen QuantiTect #QT00021147		
GHR (UPL)	TGCTTTTTCTGGAAGTGAGGA	GGTTCTTTGTACCATGATGAACCT	59
EGFR^b^	ACCTGTGCCATCCAAACTG	ACCACCAGCAGCAAGAG	
EGFR (UPL)	CATGTCGATGGACTTCCAGA	GGGACAGCTTGGATCACACT	44
SOCS2^c^	Qiagen QuantiTect #QT00079352		
SOCS2 (UPL)	GGAGCTCGGTCAGACAGG	GTTCCTTCTGGTGCCTCTTTT	60
ALAS1	TGCAGTCCTCAGCGCAGT	TGGCCCCAACTTCCATCAT	
PPIA (UPL)	ATGCTGGACCCAACACAAAT	TCTTTCACTTTGCCAAACACC	48
WT‐GHR expr. vector	GATCCACCCATTGCCCTCAA	CACCTCACTGAACTCGCCAT	
CA‐GHR (rabbit) expr. vector	AGGATGACGACTCTGGACGA	GTGCAGCCTGAAGAGTGGAT	
GH1 (UPL)	CCAACAGGGAGGAAACACAA	GACACTCCTGAGGAACTGCAC	19

^a^Growth hormone receptor.

^b^Epidermal growth factor receptor.

^c^Suppressor of cytokine signalling 2.

### 4.8 Western blot

Cells were harvested and total protein extraction was done using RIPA buffer (Pierce, Brebieres, France) supplemented with EDTA and phosphatase and protease inhibitor cocktail (Pierce). Equal amounts of protein (30–60 μg) were run on 4%–12% Bis‐Tris gels (Life Technologies) at 150 V. Proteins were transferred on nitrocellulose membranes (Sigma) by liquid transfer at 300 mA or using a BioRad semi dry transferring device at 110 V for 1 h. Fixation sites were blocked overnight with SuperBlock Blocking Buffer in tris‐buffered saline (TBS) (Pierce), and the blots were incubated with primary antibodies overnight, and secondary antibodies for 2 h. Blots were scanned and quantified on the Odyssey CLx (Science‐Tec). Quantification values were normalized to the corresponding β‐actin band. Each western blot figure is a representative of three independent experiments, and the numbers below each band represent the quantification value of a representative experiment.

### 4.9 Antibodies used for western blots and immunostaining

For western blotting, the following primary antibodies were used: phosphorylated‐STAT5 (Cell Signaling Technology Cat# 4322S, RRID:AB_10544692), STAT5 (Cell Signaling Technology Cat# 9358, RRID:AB_659905) and β‐actin (Cell Signaling Technology Cat# 3700, RRID:AB_2242334). The secondary antibodies used were Odyssey IRDye goat anti‐rabbit (LI‐COR Biosciences Cat# 926–68071, RRID: AB_10956166) or mouse (LI‐COR Biosciences Cat# 926‐32210, RRID:AB_621842) secondary antibodies (Eurobio, Courtaboeuf, France) diluted 1:5000 in SuperBlock Blocking Buffer in TBS (Pierce).

For mice brain sections, sections were fixed by acetone/methanol 1:1 prior to incubation with primary and secondary antibodies and counterstained with 4′,6‐diamidino‐2‐phenylindole (DAPI) (Thermo Scientific). Sections were stained with Mayers's hematoxylin (#MHS32, Sigma) and eosin (mix of eosin Y #341972Q and Orange G #1.15925.0025, VWR, Fontenay‐sous‐Bois, France). Sections were stained with anti‐NuMA primary antibody (Thermo Fisher Scientific Cat# PA5‐22285, RRID:AB_11157241) with secondary antibody goat anti‐rabbit (Thermo Fisher Scientific Cat# A‐11034, RRID:AB_2576217). For the cell invasion analysis in mice brains, NuMA‐positive cells were quantified on coronal sections throughout entire brains using the QuPath cell detection software.

For human FFPE sections, slides were deparaffinized with xylene, ethanol 100%, ethanol 90%, ethanol 70%, ethanol 50% and water baths. Antigens were retrieved by microwave heating (power 400 W) and blocked with 5% BSA, 5% goat serum in phosphate saline buffer (PBS) in .2‐M triton. Sections were stained with anti‐pSTAT5 primary antibody (Cell Signaling Technologies #C11C5, RRID: AB_823649) and the anti‐GHR_cyt‐mAb_ monoclonal antibody directed against the intracellular domain of GHR.^28^ Vector Elite ABC HRP kit with DAB substrate (Vector Laboratories, Burlingame, CA, USA) was used for detection of IHC slides, with hematoxylin as counterstain.

### 4.10 ELISA for GH in plasma and cell supernatant

Plasma GH levels of patients from ONT tissue bank for whom tumour levels of GHR mRNA had been determined were measured using ELISA (DGH00, R&D Systems, Abingdon, UK). The same assay was used to measure GH secreted by GBM cell cultures. Briefly, 500 000 cells were plated in 6‐well plates in serum‐free B27/GF/FGF‐containing DMEM/F12 medium. Forty‐five hours later, cell suspension (neurospheres) was harvested, centrifuged, and supernatant was isolated and frozen until use.

### 4.11 GHR, GH, luciferase and CRISPR/Cas9 expression vectors

Expression vectors for human WT and rabbit CA‐GHR were generous gifts from Mike Waters and Andrew J. Brooks (University of Queensland, Australia).^33^, ^34^ The CA‐GHR vector was generated by removing the extracellular domain of rabbit *GHR* (to ensure the absence of human GH stimulation) and fusing the transmembrane and cytoplasmic domains to Jun zippers to achieve GH‐independent forced functional dimerization. WT‐GH vector was built using GH1 variant 1 sequence accession number NM_000515.5. The control GFP vector was obtained from Addgene.^75^ Luciferase/mKate2 vector was previously described.^76^ These vectors were then inserted into lentiviral vectors (see *Lentiviral production*). Stable transduced PDCLs were generated by transduction using a multiplicity of infection of 10 and selection with puromycin for 5 days for GHR and GH vectors, or by FACS sorting for luciferase/mKate2 vectors (BioRad S3e Cell Sorter).

For CRISPR/Cas9‐mediated *GHR* knockdown, cells were transfected with .6‐μg/ml DharmaFECT, .025‐μM TracrRNA, 2‐μg/ml mKate‐Cas9 expression vector (#T‐2010‐01, U‐002000‐120, U‐004100‐120, respectively, from GE Dharmacon, CO, USA) together with .025‐μM *GHR* exon 3‐targeting crRNA (TGCCAGAGATCCATACCTGT *AGG*, GE Dharmacon) or non‐targeting crRNA (#U‐007501‐01‐05 from GE Dharmacon) according to manufacturer's instructions. Seventy‐two hours later, mKate‐Cas9‐positive single cells were isolated by FACS (BD FACSAria II, BD Biosciences, San Jose, USA) and amplified to generate a monoclonal cell population. Sequencing of exon 3 of *GHR* (forward primer: TACTGAAGCTGTGCATGGGG, reverse primer: CCTGAGAACAAGAGACCTGGC) was outsourced to GATC (Constance, Germany).

### 4.12 Lentiviral production

For all vectors, lentiviral particles were produced by transient co‐transfection of HEK 293T/17 cells (ATCC No. CRL‐11268, constitutively express the simian virus 40 large T antigen and derived from clone 17 selected specifically for its high transfectability) with the lentiviral recombinant vector carrying the transgene of interest, an encapsidation plasmid (p8.9) and a VSV envelope expressing plasmid (pVSV‐G), as previously described.^77^ Briefly, calcium phosphate co‐precipitation of plasmids was used during transfection, 48‐h post‐transfection supernatants were harvested, clarified and treated with DNAse I (Sigma‐Aldrich) prior to ultracentrifugation (60 000 *g*, +4°C), and the resulting pellet was resuspended in PBS, separated into small aliquots and frozen at −80°C until use. For GHR expression vectors, titration was performed by monitoring the amount of p24 capsid protein with the HIV‐1 p24 antigen ELISA (Helvetica Health Care). For luciferase/mKate2 and GH expression vector, titration was performed following proviral DNA genomes measurement protocol described before.^78^

### 4.13 Recombinant GH and GH‐G120K

Recombinant GH‐G120K (a competitive GHR antagonist^79^, ^80^) was produced in *Escherichia coli* and purified by ion exchange chromatography as earlier described.^81^ Its antagonistic properties were assessed using routine GHR‐dependent in vitro cell bioassays^74^ before their use in GBM PDCL models. GH (H5916, Sigma) or GH‐G120K were added directly to the full culture medium.

### 4.14 SOCS2 promoter methylation

SOCS2 promoter methylation was assessed in the panel of PDCLs following a previously described method (Base‐specific cleavage and Matrix‐Assisted Laser Desorption/Ionization Time‐of‐Flight Mass Spectrometry – MALDI‐TOF MS – assay^32^) and outsourced to Varionostic (Ulm Germany). In the GBM sample panel, promoter methylation was measured by pyrosequencing by the Epigénomique Fonctionnelle team from Université de Paris. Pyrosequencing primers were designed using the PyroMark Assay Design Software 2.0 (Qiagen). An amount of 500‐ng genomic DNA was subjected to bisulfite conversion using an EpiTect Bisulfite Kit (Qiagen, Cat# 59124). PCR reactions (list of primers in Table 2) were performed in a final volume of 25 μl, using a PyroMark PCR kit (Qiagen, Cat# 978703), with one of the primers biotinylated and containing 12.5 ng of bisulfite‐treated DNA. The initial denaturation/activation step was performed at 95°C, 15 min, followed by 50 cycles of 30 s at 94°C, 30 s at 54°C, 45 s at 72°C and a final extension step at 72°C for 10 min. The quality and the size of the PCR products were evaluated by running 5 μl of each PCR product on 1.5% (w/v) agarose gel in a .5× TBE buffer. Biotinylated PCR products (20 μl) were immobilized on Streptavidin‐coated Sepharose beads (GE Healthcare, 17‐5113‐01). DNA strands were separated using the PyroMark Q24 Vacuum Workstation, and the biotinylated single strands were annealed with .375‐μM sequencing primer and used as a template for pyrosequencing. Pyrosequencing was performed using PyroMark Q24 Advanced (Qiagen, Cat# 9002270) according to the manufacturer's instructions, and data about methylation at each CpG were extracted and analysed using the PyroMark Q24 Advanced 3.0.0 software (Qiagen).

**TABLE 2:** Primers for suppressor of cytokine signalling 2 (SOCS2) promoter methylation pyrosequencing

	Sequence	Size (bp)	Annealing Temp
Forward Region 1	TTTAGGATTTGGGGAGAAAGAGTT	315	57°C
Biotinylated‐Reverse 1	Biotin‐ACTCCCTACCTATCTAACC		
SOCS2 SEQ 1‐1	ATTTTTTTTTTTTTTGTTATTATTT	6 CGs (CG1‐6)	
Sequence to analyse	CGGACACCCCGCAGGGACTCGTTTTGGGATTCGCACTGACTTCAAGGAAGGACGCG		
SOCS2 SEQ 1–2	GAATTTTTTTTTGATTTTAG	9 CGs (CG7‐15)	
Sequence to analyse	CTCGGGCGGCCACCTGTCTTTGCCGCGGTGACCCTTCTCTCATGACCCTGCGGTGCCTTGAGCCCTCCGGGAATGGCGGGGAAGGGACGCGGA		
SOCS2 SEQ 1–3	GGGAAGAGGAGTTAGTGGG	11 CGs (CG16‐26)	
Sequence to analyse	GGACCGCGGGGTCGGCGGAGGAGCCATCCCCGCAGGCGGCGCGTCTGGCGAAGGCCCTGCGGGAGCTCG		
Forward Region 2	GGGAGTTAGGTTAGATAGGTAGGG	214	57°C
Biotinylated‐Reverse 2	Biotin‐CCTAAATCCCTAAAAAACCACTTT		
SOCS2 SEQ 2‐1	GTTAGATAGGTAGGGAG	11 CGs (CG27‐36)	
Sequence to analyse	CCGATCGGCCGCGACGCGTGCGGGAGGGAGCGCCTCCCCAAGGAAGCAGCTAGGAAGCGGGGTCGAG		
SOCS2 SEQ 2‐2	GTGGGAAGTAAAGAATAAG	6 CGs (CG37‐42)	
Sequence to analyse	ATGGAAATACGTCCCTTGCTTCCAAGGGACCGCGGAGAGCACGCTCGCAGGGTCCTGGGTCCTTGGGAATGCGTAA		

### 4.15 Cell viability, migration and invasion

All in vitro tests were performed in at least three independent experiments. For the proliferation assay, 96‐well plates were coated with 10‐μg/ml laminin (#L2020, Sigma‐Aldrich, Saint‐Quentin‐Fallavier) at 37°C for 1 h. Three thousand cells/well were then plated in full culture medium. Twenty‐four and ninety‐six hours after cell plating, cell viability was assessed using WST‐1 reagent (Roche) or CyQUANT (Thermo Fisher Scientific) according to the manufacturer's instructions. For the migration assay, spheres were plated in laminin coated 96‐well plates, in the absence or in the presence of 20‐μg/ml GH‐G120K or 1‐μg/ml RGD peptide (SelleckChem), doses that were confirmed to have no impact on cell proliferation, and individual spheres were photographed 24 h after plating. For the invasion assay, spheres were included in the extra‐cellular matrix provided in Cultrex 3‐D Basement Membrane Extract Spheroid Cell Invasion Assay (Trevigen) according to manufacturer's instructions and photographed 24 h after plating. Sphere area or spreading was quantified by ImageJ software.

### 4.16 SILAC labelling and proteomic analysis

A SILAC‐based proteomic analysis was carried out to accurately quantify the proteomes of three constructs‐transduced PDCLs (4339 GFP, 4339 WT‐GHR, 4339 CA‐GHR) derived from 4339 PDCL. All cell populations were metabolically encoded by a 3‐week exposure to one of the following SILAC labelling included in the culture medium: (1) light labelling: light l‐arginine and l‐lysine; (2) medium labelling: l‐arginine–HCl, ^13^C_6_ + l‐lysine–2HCl, 4,4,5,5‐d_4_; (3) heavy labelling: l‐Arginine–HCl, ^13^C_6_, ^15^N_4_ + l‐lysine–2HCl, ^13^C_6_, ^15^N_2_ (Thermo Fisher Scientific). For each of the three replicates, labelling was inverted for each cell line variant. All three cell populations (heavy, medium and light) were mixed before proteomic analysis, lysed with RIPA buffer supplemented with phosphatase and protease inhibitor cocktail (Pierce, Brebieres, France), separated on SDS‐PAGE gels and analysed by nano‐LC‐MS/MS (see *Proteomic data acquisition* [*Institut Curie Series*]). The mass spectrometry proteomics data have been deposited to the ProteomeXchange Consortium via the PRIDE^35^ partner repository with the dataset identifier PXD004969. Main findings from this series (Institut Curie, Paris, France) were also observed and verified in an independent mass spectrometry acquisition (see *Proteomic data acquisition* [*Yale University Serie*]).

### 4.17 Proteomic data acquisition (Institut Curie Series)

Protein extracts separated on SDS–PAGE gels (10%, Invitrogen at 30 mA during 1 h 30 min) were stained with colloidal blue staining (LabSafe GEL Blue G Biosciences). Gel slices were then excised (12 bands), and proteins were reduced with 10‐mM DTT prior to alkylation with 55‐mM iodoacetamide. After washing and shrinking the gel pieces with 100% MeCN, in‐gel digestion was performed using trypsin (Promega) overnight in 25‐mM NH_4_HCO_3_ at 30°C.

Peptides were extracted from gel slices and analysed by nano‐LC–MS/MS using an RSLCnano system (Ultimate 3000, Thermo Scientific) coupled to an Orbitrap Fusion mass spectrometer (Q‐OT‐qIT, Thermo Fisher Scientific). Samples were loaded on a C18 precolumn (300‐μm inner diameter × 5 mm; Thermo Scientific) at 20 μl/min in 2% MeCN, .1% CH_2_O_2_. After a desalting for 3 min, the precolumn was switched on the C18 column (75 μm i.d. × 50 cm, packed with C18 PepMap, 3 μm, 100 Å; Thermo Scientific) equilibrated in solvent A (2% MeCN, .1% CH_2_O_2_). Bound peptides were eluted using a 163‐min multistep linear gradient (from 1% to 6% [v/v] in 1 min, from 6% to 9% in 18 min, from 9% to 32% in 132 min and from 32% to 40% in 9 min) of solvent B (80% MeCN, .085% CH_2_O_2_) at a 400‐nl/min flow rate and an oven temperature of 40°C Survey MS scans were acquired in the Orbitrap on the 400–1500 *m*/*z* range with the resolution set to a value of 120 000 and a 4 × 105 ion count target. Each scan was recalibrated in real time by co‐injecting an internal standard from ambient air (445.12003 *m*/*z*) into the C‐trap. Tandem MS was performed by isolation at 1.6 Th with the quadrupole, HCD fragmentation with normalized collision energy of 35 and rapid scan MS analysis in the ion trap. The MS2 ion count target was set to 104 and the max injection time was 100 ms and only those precursors with charge state 2–7 were sampled for MS2. The dynamic exclusion duration was set to 60 s with a 10‐ppm tolerance around the selected precursor and its isotopes. The instrument was run in top speed mode with 3‐s cycles.

Data were acquired using the Xcalibur software (v 3.0) and the resulting spectra were interrogated by Sequest HT through Proteome Discoverer (v 1.4, Thermo Scientific) with the SwissProt Homo Sapiens database (032015). Carbamidomethyl cysteine, oxidation of methionine, N‐terminal acetylation, heavy ^13^C_6_^15^N_2_‐lysine (Lys8) and ^13^C_6_^15^N_4_‐arginine (Arg10) and medium ^2^H_4_‐lysine (Lys4) and ^13^C_6_‐arginine (Arg6) were set as variable modifications. Specificity of digestion was set for trypsin and allowed two missed cleavage sites. Mass tolerances in MS and MS/MS were set to 10 ppm and .5 Da, respectively. The resulting files were further processed using myProMS.^82^ The Sequest HT target and decoy search result were validated at 1% false discovery rate (FDR) with Percolator. For SILAC‐based protein quantification, peptides XICs (Extracted Ion Chromatograms) were retrieved from Proteome Discoverer. Scale normalization was applied to compensate for mixing errors of the different SILAC cultures. Protein ratios were computed as the geometrical mean of related peptides ions ratios using the R package limma^83^ and *p*‐values were adjusted using Benjamini–Hochberg FDR control threshold set to .05. Analysis of biological replicates of all the samples identified a total of 9535 proteins at an FDR of 1% (5639 common proteins). Of the 8305 quantified proteins (WT/GFP and CA/GFP), 3188 of them were quantified with at least three peptides, the latter of which were used for all subsequent analysis in this study.

### 4.18 Proteomic data acquisition (Yale University Serie)

Proteins were precipitated by Chloroform:MeOH:water (1:4:4) for shipping. Protein pellet was washed twice with methanol then vacuum dried briefly and reconstituted in a urea:ammonium buffer (8 M:.4 M), digested with Lys‐C (overnight) followed by trypsin (Promega, Inc.; for 5 h) and desalted using an RP macro‐spin (The Nest Group) desalting cartridge. The eluted peptides were reconstituted in .1% formic acid prior to injection onto an LC–MS/MS mass spectrometer. LC–MS/MS was performed on an LTQ Orbitrap Elite (Thermo Fisher Scientific) equipped with a Waters Symmetry C18 (180 μm × 20 mm) trap column and a 1.7 μm, 75 μm × 250 mm nanoAcquity UPLC column (35°C). Trapping was done using 99% Buffer A (100% water, .1% formic acid) and peptide separation was accomplished using a linear gradient of solvents A (.1% Formic Acid in Water) and B (.075% formic acid in acetonitrile) over 210 min, at a flow rate of 300 nl/min. MS spectra were acquired in the Orbitrap using 1 microscan and a maximum injection time of 900 ms followed by three data dependent MS/MS acquisitions in the ion trap (with precursor ions threshold of >3000). The total cycle time for both MS and MS/MS acquisitions was 2.4 s. Peaks targeted for MS/MS fragmentation by collision induced dissociation were first isolated with a 2‐Da window followed by normalized collision energy of 35%. Dynamic exclusion was activated where former target ions were excluded for 30 s. Data were processed and analysed as previously described in myProMS.^82^

### 4.19 Orthotopic GBM in vivo model

Transduced GBM cells (4339^GFP^, 4339^WT‐GHR^, 4339^CA (constitutively activated)‐GHR^, N14‐1525^GFP^, N14‐1525^WT‐GH^, N13‐1520^GFP^ and N13‐1520^CA‐GHR^) were implanted (1.4 × 10^5^ cells/2 μl) into the brain of Nude Hsd:Athymic Nude‐Foxn1nu mice (Envigo, 8‐week old females, 3 or 12 animals/group) by stereotaxic injection at Bregma AP: +.1; ML: −.15; DV: −.25 under isoflurane anaesthesia and following ethically approved protocol #17503 2018111214011311 v5. Tumour growth was monitored by bioluminescence imaging following 100‐μl luciferin (Perkin Elmer) subcutaneous injection at 30 mg/ml, and image acquisition with an IVIS Spectrum imager (Perkin Elmer). Tumour take was evaluated by determining the day when bioluminescence signal was multiplied 10‐fold compared to the first bioluminescence measured 8‐day post‐graft. When animals reached ethical endpoints, they were sacrificed and the date was recorded. Mice were sacrificed by anaesthesia followed by dislocation 4 months after inoculation, and their brain was harvested, frozen and sectioned using a cryostat.

### 4.20 Study approval

All protocols involving work with live animals were reviewed and approved by the local ethical committee and the Ministère de l'Enseignement Supérieur et de la Recherche de France (project 17503 2018111214011311). GBM tissue samples and clinical annotations were provided by the neuropathology laboratory of Pitié‐Salpêtrière University Hospital and obtained as part of routine resections from patients under their informed consent (ethical approval number AC‐2013‐1962).

### 4.21 Statistics

A paired‐sample Wilcoxon test was computed between GHR expression versus EGFR or SOCS2 expression data extracted from publicly available datasets.^9^, ^13^, ^18^, ^19^, ^20^ Pearson's chi‐square tests were performed to compare molecular subgroups and gene alterations distribution between GHR^high^ and GHR^low^ GBMs in the TCGA dataset. Both analyses were performed on R. All other statistical tests were performed using GraphPad Prism 6. Fisher's exact test was performed to compare age at diagnostic and sex ratio in the ONT series. OS curves were generated using the Kaplan–Meier method and compared with a log‐rank or Gehan–Breslow–Wilcoxon tests. Analysis for identification of signalling pathways and biological modules (*p*‐values and activation *z*‐score) from expression arrays and proteome data was performed using Ingenuity Pathway Analysis software (Qiagen).^84^ For all other analysis, one‐way ANOVA with Tukey's test for one parameter multiple comparisons, two‐way ANOVA with Sidak's test for two parameters multiple comparisons, or two‐tailed *t*‐test for single comparisons were performed on the mean ± s.e.m.

## FINANCIAL SUPPORT

This work is part of the national program Cartes d'Identité des Tumeurs^®^ (CIT) (http://cit.ligue‐cancer.net/) funded and developed by La Ligue Contre le Cancer. This work was supported by grants from:

– L'Association pour la Recherche sur les Tumeurs Cérébrales (ARTC) (AI/MV)
– La Fondation ARC pour la Recherche sur le Cancer GlioTEx project (AI)
– Cancéropôle Ile‐de France Emergence program 2015‐1‐EMERG‐17‐INSERM 6‐1 (AI/MV/VG)
– US National Institutes of Health NIDDK R01‐DK107441 (SJF)
– US Veterans Administration Merit Review 1I01BX003718 (SJF)
– FRM DGE 20121125630 (FDi, DL)
– NIH A09562 MIMED‐Neurovascular Renegeration (JLT)
– SiRIC CURAMUS grant INCa‐DGOS‐Inserm_12560 (MS)
– Program “investissements d'avenir” ANR‐10‐IAIHU‐06 (MS)
– Ligue Nationale contre le Cancer (LNCC; équipe labellisée) (MS)
– Yale School of Medicine (TTL)
– Office of The Director, National Institutes of Health (S10OD02365101A1, S10OD019967 and S10OD018034) (TTL)

## CONFLICT OF INTEREST

The authors declare that there is no conflict of interest that could be perceived as prejudicing the impartiality of the research reported.

## Supporting information

Supporting Information
