# Supplementary material for: Identification of growth hormone receptor as a relevant target for precision medicine in low‐EGFR expressing glioblastoma
Source: Clin Transl Med. 2022 Jul 8;12(7):e939. doi: 10.1002/ctm2.939 (PMC9270581; doi:10.1002/ctm2.939)

Supplementary Materials for

**Identification of growth hormone receptor as a relevant target for precision medicine in low-EGFR expressing glioblastoma**

Maïté Verreault^†^, Irma Segoviano^†^, Shai Rosenberg, Nolwenn Lemaire, Charlotte Schmitt, Jérémy Guehennec, Louis Royer-Perron, Jean-Léon Thomas, TuKiet T. Lam, Florent Dingli, Damarys Loew, François Ducray, Sophie Paris, Catherine Carpentier, Yannick Marie, Florence Laigle-Donadey, Audrey Rousseau, Natascha Pigat, Florence Boutillon, Franck Bielle, Karima Mokhtari, Stuart J. Frank, Aurelien de Reyniès, Khê Hoang-Xuan, Marc Sanson, Vincent Goffin, Ahmed Idbaih^*^

^†^co-first authors

*Corresponding authors: Ahmed Idbaih: [ahmed.idbaih@aphp.fr](mailto:ahmed.idbaih@aphp.fr) ; Maïté Verreault: maite.verreault@icm-institute.org

**This PDF file includes:**

Figs. S1 to S6

Tables S1 to S4

Fig. S1. Clinical and molecular data from the GBM cohort according to their GHR status (high vs low). (A) EGFR gene expression (median probe level) distribution and EGFR high vs low subgroup definition based on expression microarray data in the ONT cohort. Groups were defined using the median values as a cutoff. (B) Kaplan-Meier curves indicating % overall survival of patients from ONT series with GBM^GHR high^ (dashed line; n = 18) and patients with GBM^GHR low^ (continuous line; n = 36) from the ONT cohort. Analysis was performed by log-rank test. (C) Box plot indicating age in years at diagnosis of with GBM^GHR high^ patients (left; n = 18) and patients with GBM^GHR low^ (right; n = 36) from the ONT cohort. (D) Sex distribution of patients with GBM^GHR high^ patients (left; n = 18; ratio M/F of 0.56) and patients with normal GBM^GHR low^ (right; n = 36; ratio M/F of 3) from the ONT cohort. (E) Proportions of *IDH1* mutations in GBM^GHR high^ vs GBM^GHR low^ in TCGA and ONT cohorts. Proportions are normalized on 1 and were analyzed by Pearson’s Chi-square test.


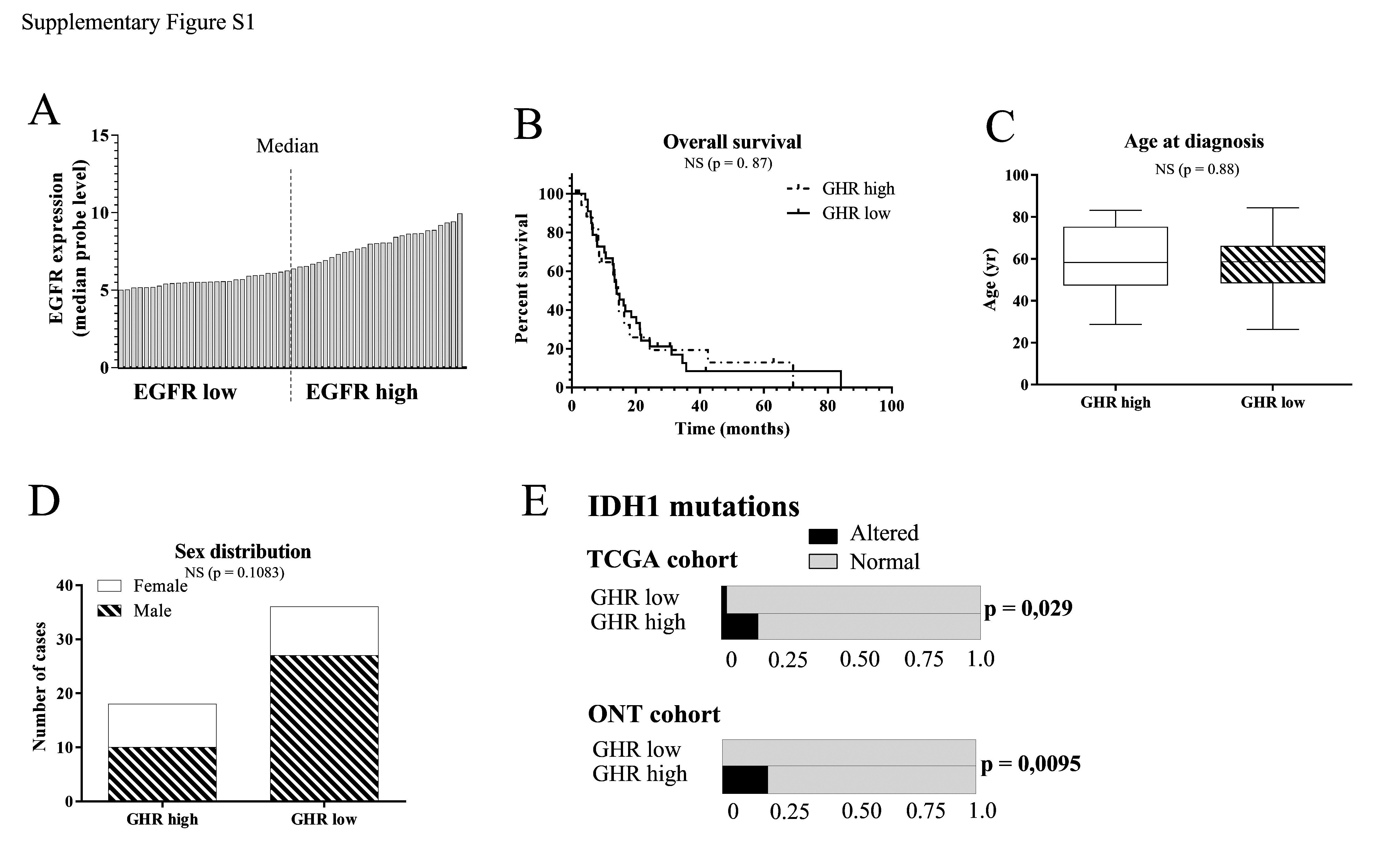


Fig. S2. Validation in independent series of GBM. Clustering of five independent series of GBMs based on the expression of EGFR, SOCS2 and GHR are consistent with our data. Indeed, in the series of (A) Philips et al. (, (B) Freije et al., (C) Sun et al., (D) CGARN et al, and (E) Brennan et al. (9, 13, 18-20), two major subtypes of GBMs are identified: i) dendrogram branch 1) EGFR^low^/SOCS2^low^/GHR^high^ GBMs and ii) dendrogram branch 2) EGFR^high^/SOCS2^high^/GHR^low^ GBMs. (F) Color code for expression heat maps.


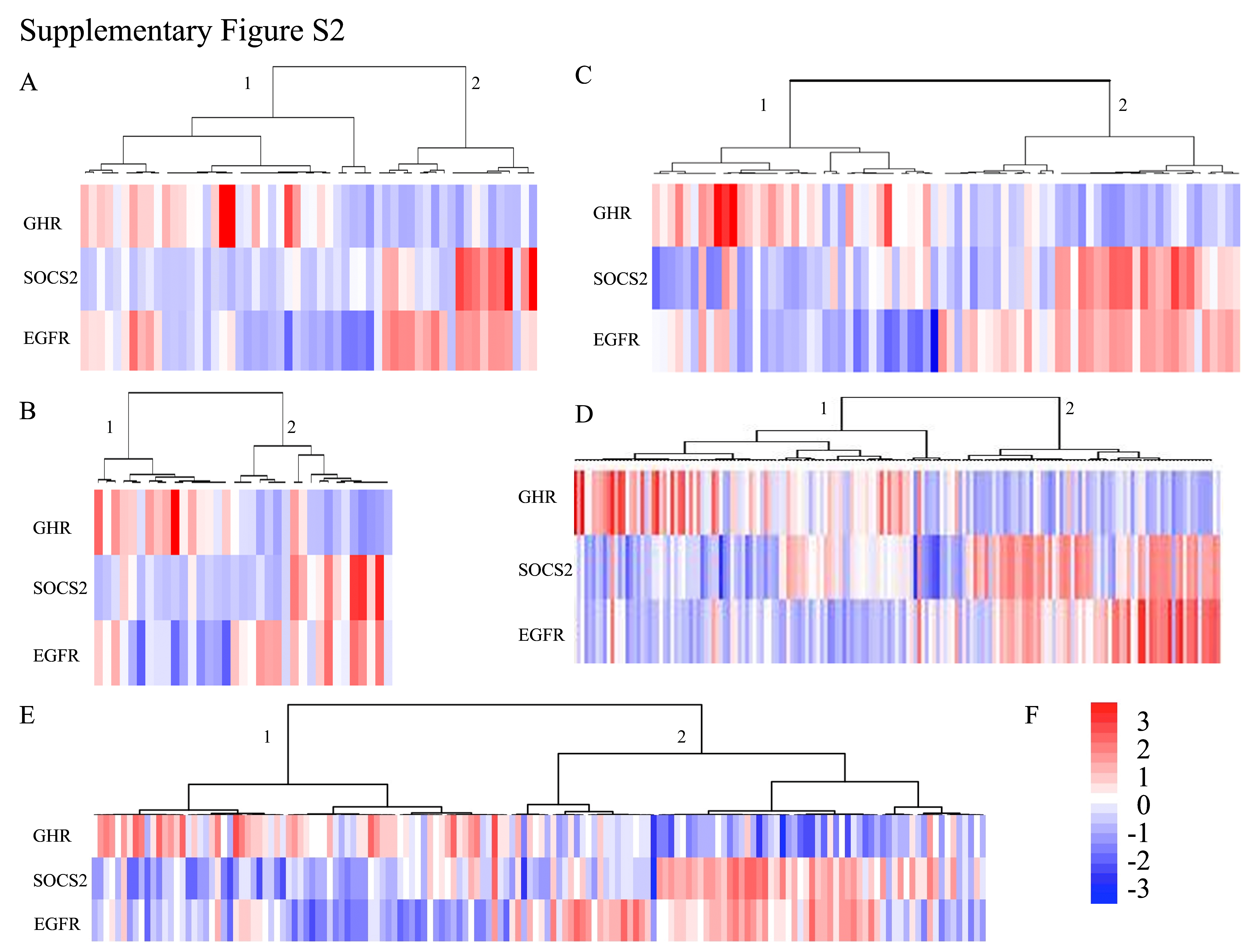


**Fig. S3. Immunohistochemistry staining of GBM tissue sections showing GHR and p-STAT5. (A)** GHR staining of three regions per cases, for two GBM^GHR high^ and two GBM^GHR low^. Staining was observed as regions of positive cells surrounded by regions of negative cells, with cases such as GHR^high^ #2 showing higher density of positive cells. (**B**) P-STAT5 staining from corresponding regions in sections harvested proximally to those shown in (A). (**C**) Negative controls performed in absence of incubation with primary antibodies, from corresponding regions in sections harvested proximally to the first sections shown in (A) and (B).

**
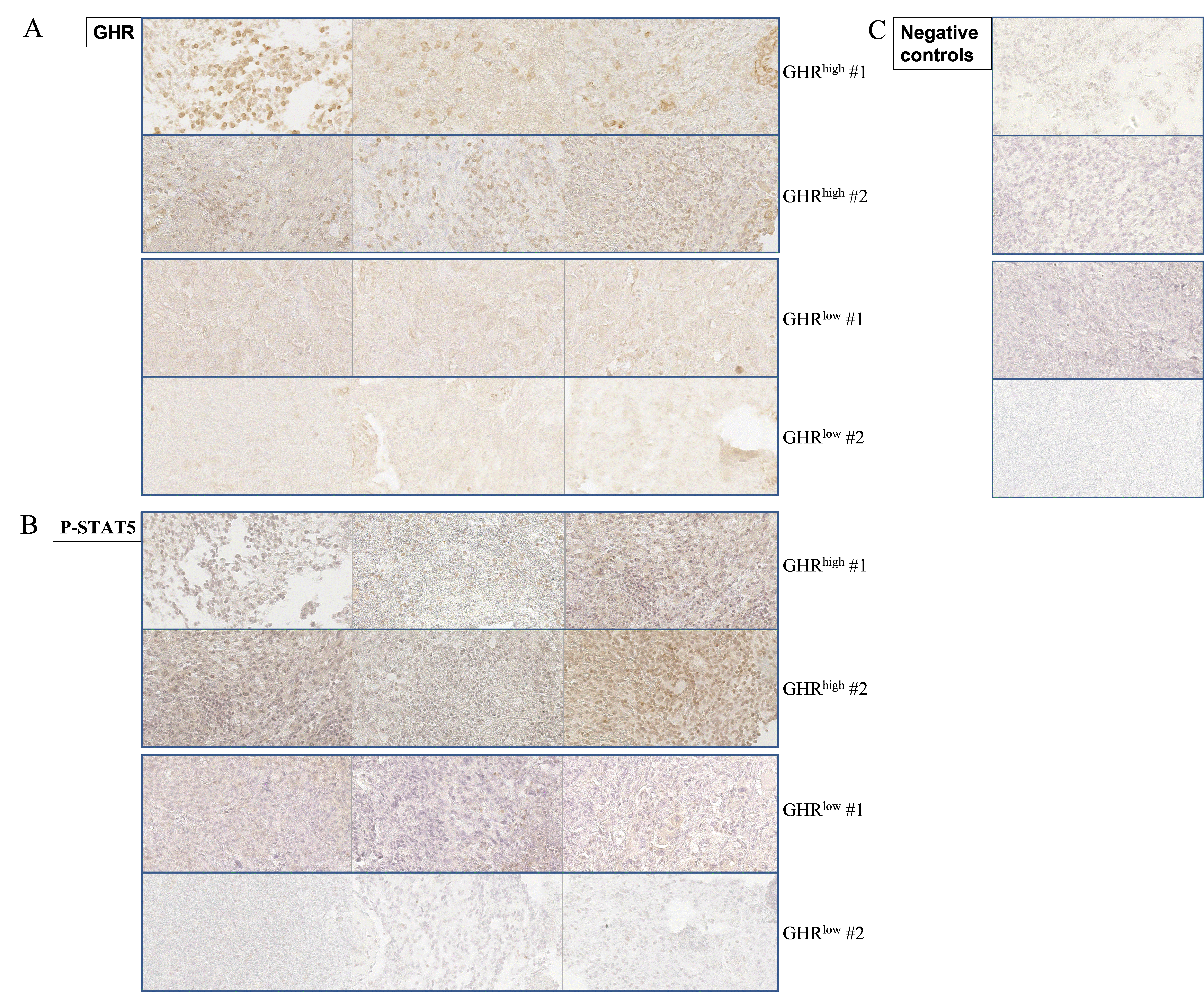
**

Fig. S4. GH/GHR mRNA levels and p-STAT5 protein levels from the genetically transformed PDCLs with the GHR/GH constructs. (A) Expression of GHR by RT-qPCR relative to 4339^GFP^ in 4339^WT-GHR^ and 4339^CA-GHR^ (n = 3). (B) Western immunoblot showing p-STAT5 (high and low exposure), STAT5 and β-actin (loading control) for 4339^GFP^, 4339^WT-GHR^ and 4339^CA-GHR^ following exposure to hGH. The level of p-STAT5 in 4339^GFP^ was undetectable in the absence or presence of hGH. Ectopic expression of GHR in 4339^WT-GHR^ enhanced GH-stimulated p-STAT5 levels compared to 4339^GFP^. As expected, constitutive p-STAT5 was observed in 4339^CA-GHR^, was higher than in 4339^GFP^ cells and it was unaffected by hGH stimulation. (C) Expression of GHR by RT-qPCR relative to N13-1520^GFP^ in N13-1520^WT-GHR^ and N13-1520^CA-GHR^ (n = 3). (D) Western immunoblot showing p-STAT5 (high and low exposure), STAT5 and β-actin (loading control) for N13-1520^GFP^, N13-1520^WT-GHR^ and N13-1520^CA-GHR^ following exposure to hGH. The level of p-STAT5 in N13-1520^GFP^ was undetectable in the absence or presence of hGH. Ectopic expression of GHR in N13-1520^WT-GHR^ enhanced GH-stimulated p-STAT5 levels compared to 4339^GFP^. As expected, constitutive p-STAT5 was observed in N13-1520^CA-GHR^, was higher than in N13-1520^GFP^ cells and it was unaffected by hGH stimulation. (E) Expression of GHR and GH by RT-qPCR relative to N14-1525^GFP^ in N14-1525^WT-GH^ (n = 3). (F) Western immunoblot showing p-STAT5 and β-actin (loading control) for N14-1525^GFP^ and N14-1525^WT-GH^ following exposure to GH. The level of p-STAT5 in N14-1525^GFP^ was undetectable in the absence of GH and increased 27-fold when exposed to GH, consistent with high endogenous GHR levels. Endogenous expression of GH in N14-1525^WT-GH^ enhanced basal p-STAT5 levels compared to N14-1525^GFP^ which were unaffected by GH stimulation. (G) Western immunoblot showing the impact of pharmacological (GH-G120K) and knockdown (KD) on p-STAT5 expression in GBM1^GHR high^. When the GBM1^GHR high^ parental cell line was exposed to increasing concentrations of GH-G120K, GH-induced p-STAT5 was reduced in a dose-dependent manner. *GHR* knockdown (KD) resulted in a 10-40% reduced level of GH-induced p-STAT5 and further amplified the impact of GH-G120K on p-STAT5 inhibition, especially at the lowest GH-G120K concentration tested.


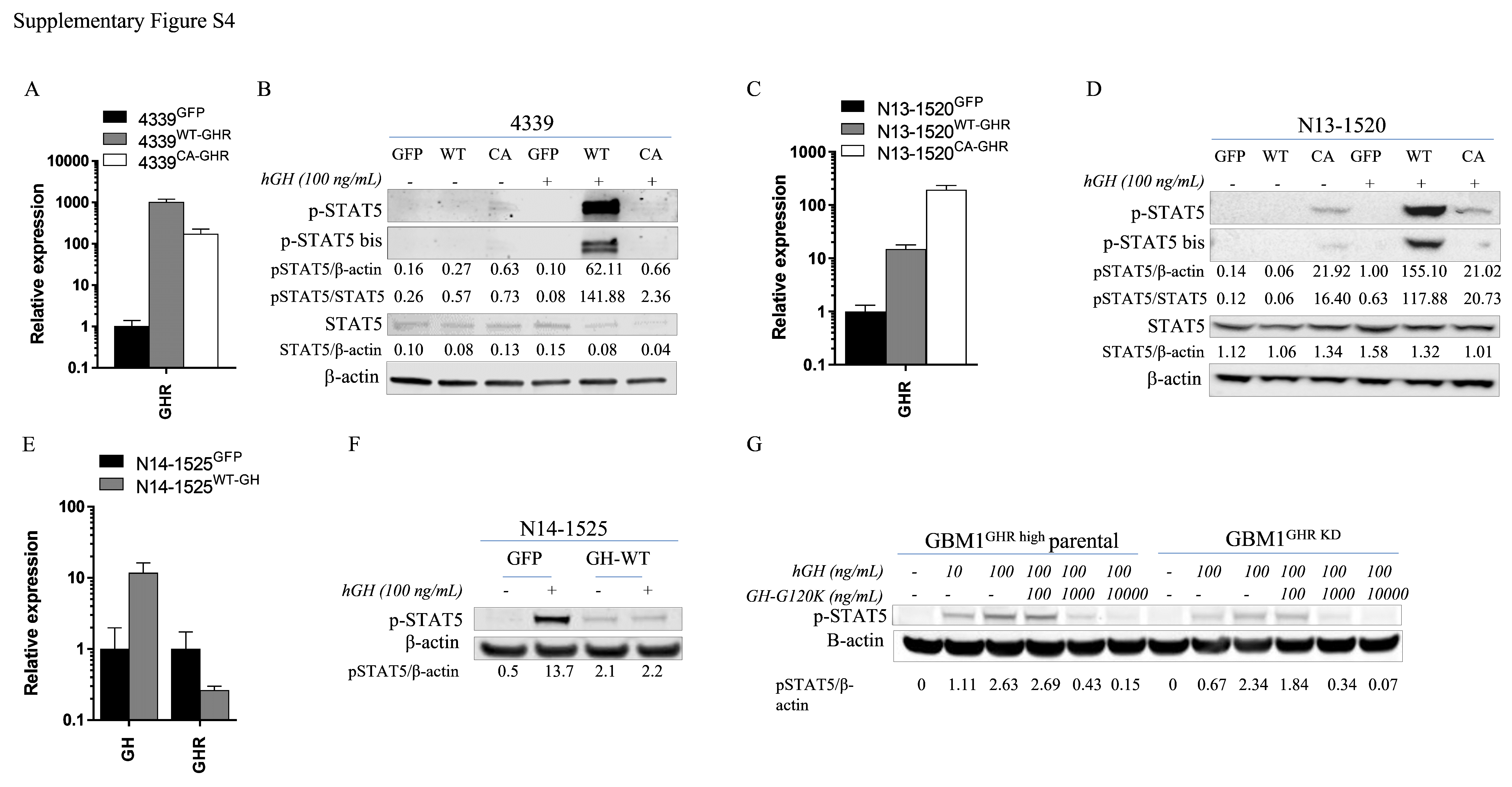


Fig. S5. Heatmap showing the top most differentially expressed proteins in 4339^WT-GHR^ or in 4339^CA-GHR^ vs 4339^GFP^ (p < 0.005, expression ratio ≥ 10 or ≤ 0.1 in either 4339^WT-GHR^ or 4339^CA-GHR^ as in Supplementary Table S3 and S4)


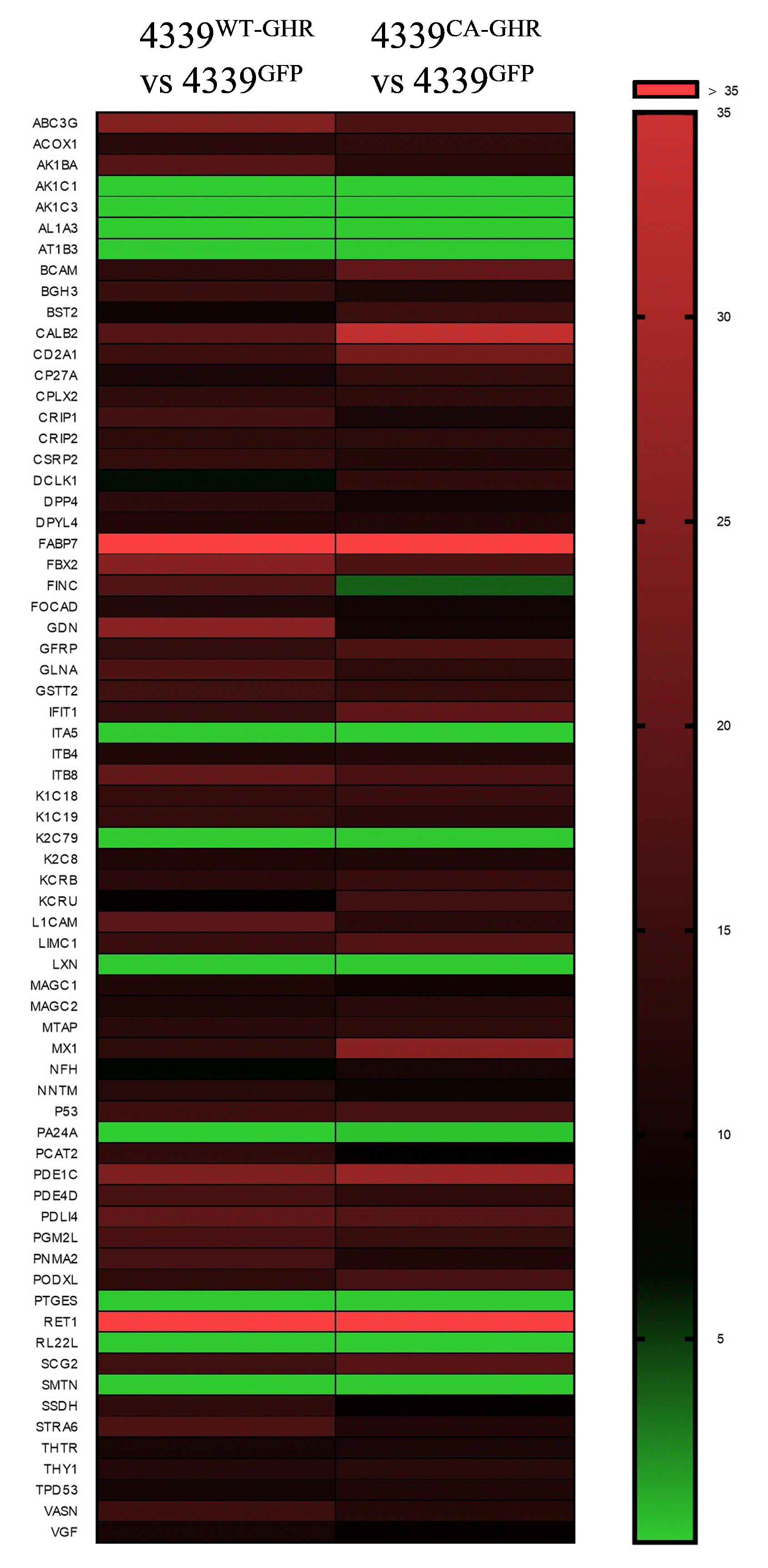


**Fig. S6.** **H&E-stained tissue sections of the parental GBM from which 4339 PDCL is derived**, showing regions with high nucleocytoplasmic ratio typical of tumors with neuronal primitive component. Black arrowhead: undifferentiated tumor cells with scant cytoplasm, do not express GFAP, and with a MKI67 proliferation index of 70%. White arrowhead: astrocytic tumor cell with large eosinophilic cytoplasm, expressing GFAP and with a MKI67 proliferation index of 10 %.

**
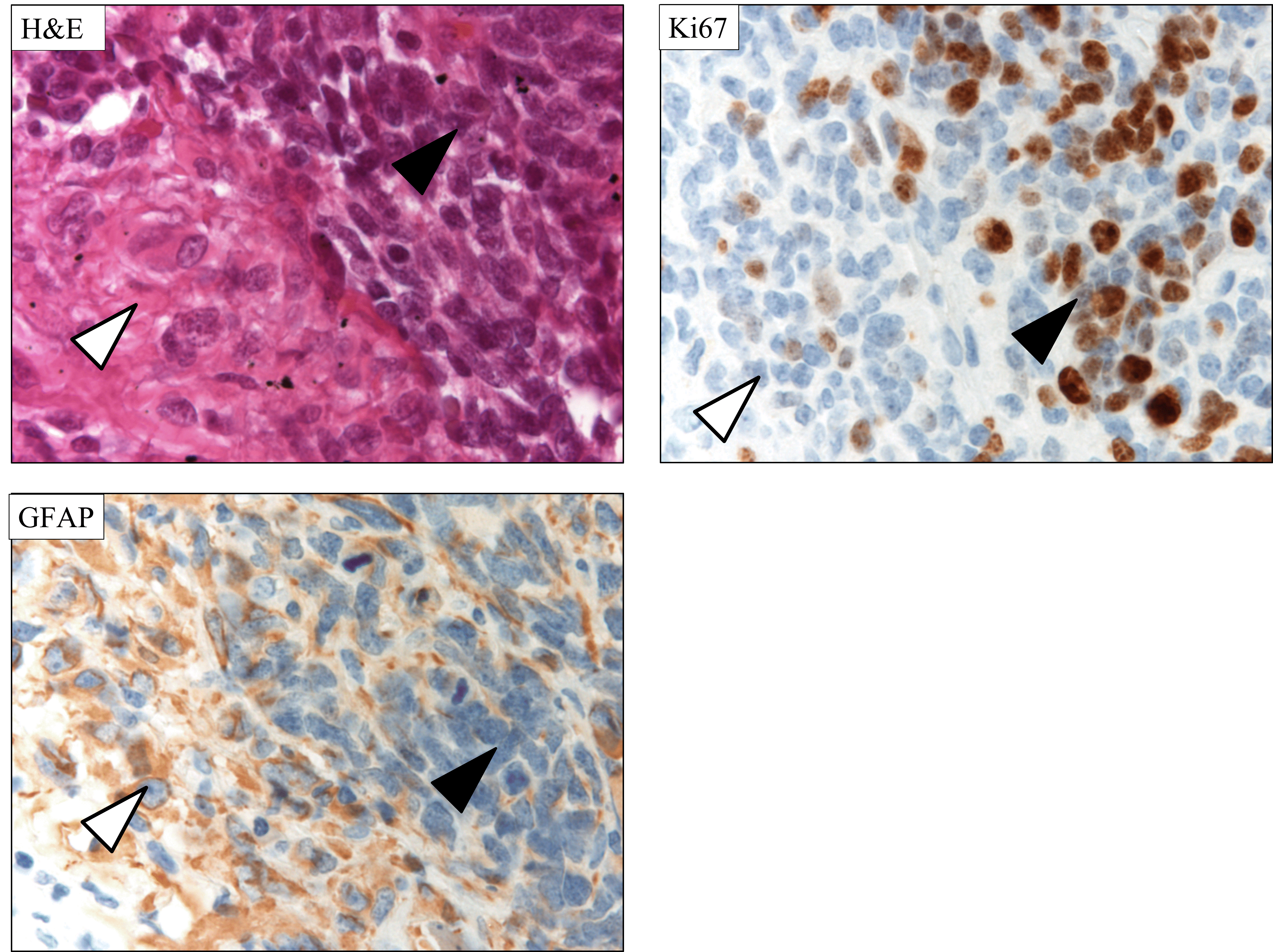
**

Table S1. Most significantly downregulated genes between low and high EGFR expressing GBM (p < 0.001)

Table S2. Most significantly upregulated genes between low and high EGFR expressing GBM (p < 0.001)

Table S2 continued. Most significantly upregulated genes between low and high EGFR expressing GBM (p < 0.001)

Table S3. Most differentially expressed proteins between 4339^WT-GHR^ vs 4339^GFP^ (p < 0.005; expression ratio ≥ 10 or ≤ 0.1)


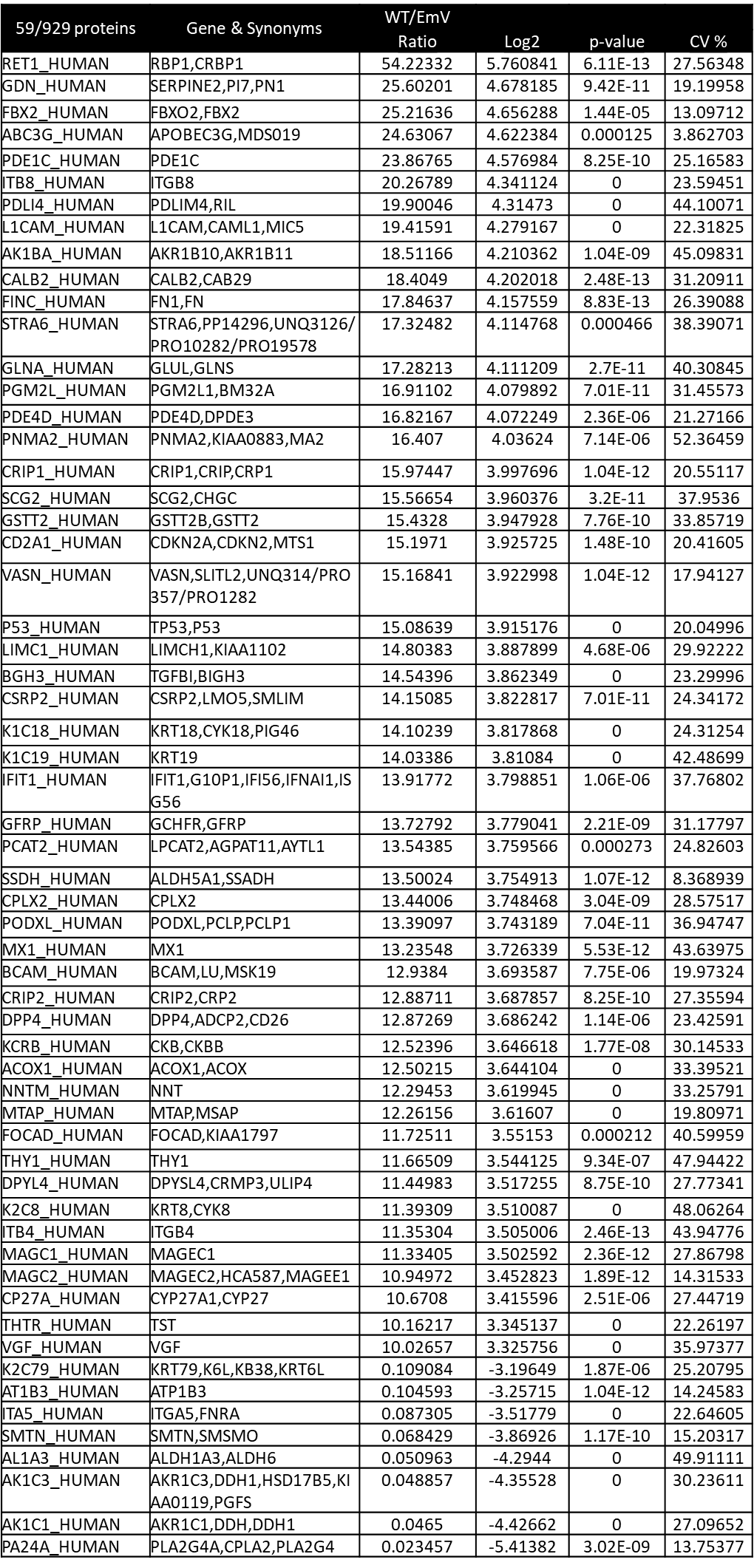


Table S4. Most differentially expressed proteins between 4339^CA-GHR^ vs 4339^GFP^ (p < 0.005; expression ratio ≥ 10 or ≤ 0.1)


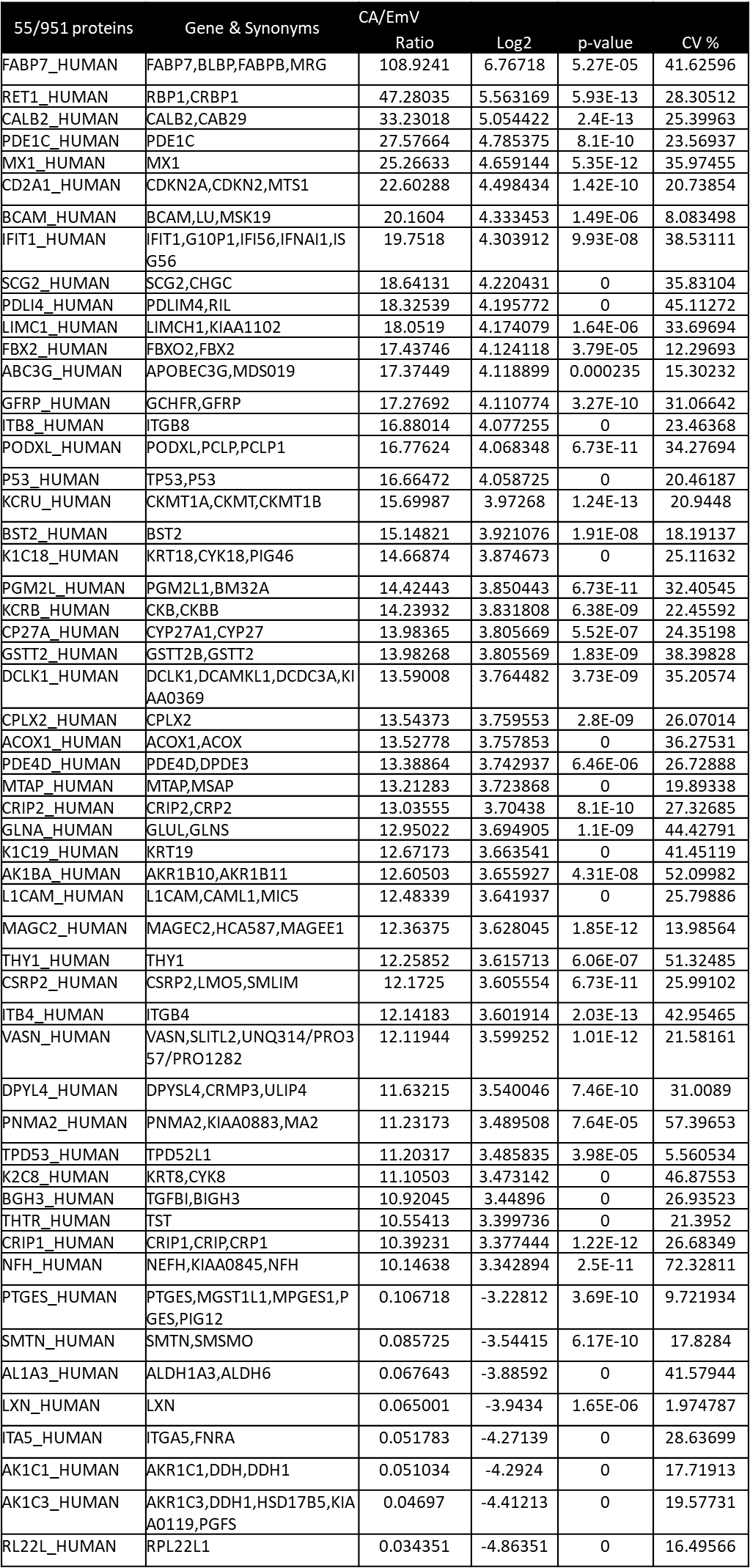

Supplement: Supplementary file 1 — Supporting Information [file CTM2-12-e939-s001.docx]
